# From iconic handshapes to grammatical contrasts: longitudinal evidence from a child homesigner

**DOI:** 10.3389/fpsyg.2014.00830

**Published:** 2014-08-21

**Authors:** Marie Coppola, Diane Brentari

**Affiliations:** ^1^Departments of Psychology and Linguistics, Language Creation Laboratory, University of ConnecticutStorrs, CT, USA; ^2^Department of Linguistics, Sign Language Laboratory, University of ChicagoChicago, IL, USA

**Keywords:** sign language, homesign, gesture, phonology, morphology, language emergence, iconicity, grammaticalization

## Abstract

Many sign languages display crosslinguistic consistencies in the use of two iconic aspects of handshape, handshape type and finger group complexity. Handshape type is used systematically in form-meaning pairings (morphology): *Handling handshapes* (Handling-HSs), representing how objects are handled, tend to be used to express events with an agent (“*hand-as-hand*” iconicity), and *Object handshapes* (Object-HSs), representing an object's size/shape, are used more often to express events without an agent (“*hand-as-object*” iconicity). Second, in the distribution of meaningless properties of form (morphophonology), Object-HSs display higher *finger group complexity* than Handling-HSs. Some adult homesigners, who have not acquired a signed or spoken language and instead use a self-generated gesture system, exhibit these two properties as well. This study illuminates the development over time of both phenomena for one child homesigner, “Julio,” age 7;4 (years; months) to 12;8. We elicited descriptions of events with and without agents to determine whether morphophonology and morphosyntax can develop without linguistic input during childhood, and whether these structures develop together or independently. Within the time period studied: (1) Julio used handshape type differently in his responses to vignettes with and without an agent; however, he did not exhibit the same pattern that was found previously in signers, adult homesigners, or gesturers: while he was highly likely to use a Handling-HS for events with an agent (82%), he was less likely to use an Object-HS for non-agentive events (49%); i.e., his productions were heavily biased toward Handling-HSs; (2) Julio exhibited higher finger group complexity in Object- than in Handling-HSs, as in the sign language and adult homesigner groups previously studied; and (3) these two dimensions of language developed independently, with phonological structure showing a sign language-like pattern at an earlier age than morphosyntactic structure. We conclude that iconicity alone is not sufficient to explain the development of linguistic structure in homesign systems. Linguistic input is not required for some aspects of phonological structure to emerge in childhood, and while linguistic input is not required for morphology either, it takes time to emerge in homesign.

## Introduction

Striking cross-linguistic similarities have been described in how sign languages use handshape to mark linguistic distinctions; see, e.g., Brentari et al. (2012) for morphosyntax and Brentari and Eccarius (2010) for phonology^1^. This paper will discuss two aspects of handshapes and explore how these forms are used in a grammatical system longitudinally in a child developing a homesign system. The first is the *handshape type*, which characterizes the way that a handshape expresses a meaning. Specifically, *Handling* handshapes depict the hand manipulating an object, while *Object* handshapes capture an object's properties by using the handshape to depict the whole item, or size and shape dimensions of the item. Handling handshapes are used to describe events in which an agent manipulates an object, and Object handshapes describe events or arrays of objects that do not involve an agent. Thus, this use of different handshape classes, or types, constitutes a *morphosyntactic* distinction in sign languages. The second dimension of handshape is selected finger group complexity, which involves the selection of phonological groups of fingers. Selected finger groups with higher complexity^2^ are associated with Object handshapes, and finger groups with lower complexity are associated with Handling handshapes. This use of a meaningless property of handshape nested within the above-mentioned morphological contrast results in a *morphophonological* distinction in sign languages.

The present study connects to several of the themes of this special issue: specifically, in the way that meaning in natural languages and its phonological vessels (in this case, manual gestures) interact. In particular, iconicity has been proposed as a likely, or even inevitable resource that can be tapped in the processing of sign languages (Vigliocco et al., 2005), in the acquisition of structure in sign languages (Ormel et al., 2009; Thompson et al., 2009), in the emergence of linguistic structure in new languages (Meir et al., 2007), in the very organization of sign language grammars (Cuxac, 1999; Demey and van der Kooij, 2008; Meir, 2010), and as a general property of both signed and spoken languages (Perniss et al., 2010). In the realm of experimental semiotics, Fay et al. (2013, 2014) argue that gesture is likely to bootstrap human communication systems in the absence of linguistic input precisely because it affords greater iconicity than the auditory modality. We will argue that iconicity is a multilayered, complex notion that must be treated with care, especially when evaluating its influence on the distribution of linguistic components in grammatical systems.

Accessibility to iconicity in development does not happen all at once in sign language or gesture. For example, Brentari et al. (2012) have described *hand-as-hand* iconicity in Handling handshapes as distinct from *hand-as-object* iconicity in Object handshapes when gesturers and users of conventional sign languages describe events; Padden et al. (2013) apply a similar distinction in their work as well. The level of accessibility to different kinds of iconicity depends on the ambient language, the age and life experience of the participant, as well as the nature of the task. The handling of objects is a human action, argued to be easier to produce in gesture than a static object or action by an object (Piaget, 1952; Werner and Kaplan, 1963). On the other hand, studies of child gesture show that Object handshapes are used before Handling handshapes (Kaplan, 1968; Overton and Jackson, 1973; Boyatzis and Watson, 1993; O'Reilly, 1995; Tomasello et al., 1999). Namy et al. (2004) argue that iconicity as a factor in concept acquisition is not immediately available to infants and toddlers, and takes time to learn. Moreover, in all of these studies one must also ask whether the experimental task itself was designed to address specific minimal differences in meaning that can be expressed in componential form; for example, the two properties of handshape relevant here are elicited using a vignette description task which targets minimal differences in handshape meaning, while carefully controlling for location and movement.

Both morphosyntax and morphophonology use iconicity in different and independent ways, which will be described in the next two sections. Moreover, while the importance of iconicity as a source of raw material for new forms in sign languages cannot be ignored, the *distribution* of these elements is abstract and can also be arbitrary: *handshape type* pertains to morphosyntactic representation while *selected finger group complexity* is at the level of morphophonological representation (Brentari, 2007, 2011). We begin with an overview of how handshape type is organized in the morphosyntax of sign languages, and then describe how finger group complexity varies systematically within those morphological handshape classes, and gives rise to morphophonological structure. In two studies, we examine the development of the uses of these aspects of handshape in one homesigning child, and use comparative analyses with other participant groups to uncover the sources of convergence between his patterns and the crosslinguistic patterns we have observed in sign languages.

### Morphosyntax in classifier constructions

**Figure 1:**
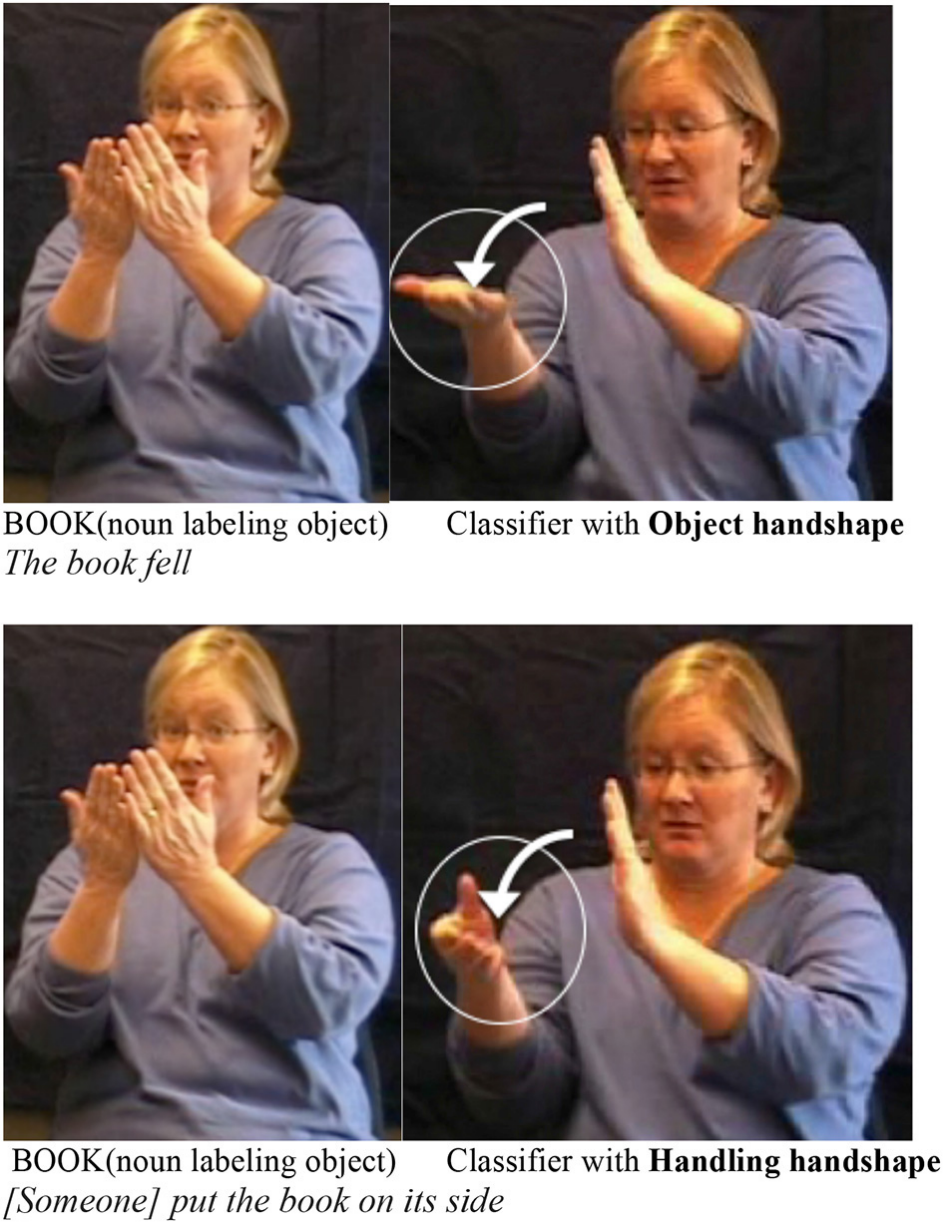
**Examples of events expressed by classifier constructions in ASL that use different handshape types: a Non-agentive/intransitive event expressed via an Object handshape (top right, circled handshape) vs. an Agentive/Transitive event expressed via a Handling handshape (bottom right, circled handshape)**. This is a minimal pair of sentential, syntactic structure and the difference in meaning arises from the difference in handshape type. The lexical item for BOOK depicted first in each example simply labels the object in the event^4^. For videos of these examples, see Coppola (2014).

Despite the apparent iconicity of Object handshapes and Handling handshapes, both types of handshapes can contribute to morphological structure. Events involving the motion and location of people and objects are preferentially described in sign languages using handshapes in particular configurations and orientations, combined with movements and locations^3^. These morphologically complex predicates are known as *classifier constructions* in the sign language literature (see Emmorey, 2003 and Zwitserlood, 2012 for summaries and examples from a variety of sign languages), and these components have been analyzed as discrete, meaningful, productive forms that are stable across related contexts (Supalla, 1982; Emmorey and Herzig, 2003; Eccarius, 2008). Our work pertains to “whole entity” and “SASS” (descriptor) classifiers (considered Object classifiers), and to “Handling” classifiers, exemplified in Figure 1. The Object handshape circled in Figure 1 (top) uses hand-as-object iconicity to represent a flat object, the book itself, involved in a “falling” event. The Handling handshape circled in in Figure 1 (bottom) uses hand-as-hand iconicity to represent someone holding/moving a book.

Benedicto and Brentari (2004) found that handshape types (Handling vs. Object) are not simply morphological forms with discrete meanings, but rather check features of argument structure within the syntactic tree. The “willing” test and the “imperative” test are widely known to detect agents crosslinguistically (Van Valin, 1993); by adding the sign WILLING or FINISH (with the negative imperative meaning “stop!”) to ASL sentences containing the classifier predicates in Figure 1, it is possible to detect the presence of an agent. Both WILLING and FINISH can be added to the sentence in Figure 1 (bottom) to obtain well-formed, grammatical sentences (English translation from ASL: “*[Someone] willingly put the book on its side*” and “*Stop putting the book on its side!*”). In contrast, adding WILLING or FINISH to the sentence in Figure 1 (top) obtains ungrammatical sentences (English translation from ASL: ^*^“The book fell *willingly*” and ^*^“Book, *stop* falling!”). Since the only part of the structures in Figure 1 that varies is the handshape, the differences obtained using these diagnostic syntactic tests is attributed to handshape, indicating that the sentence with the Handling handshape (Figure 1, bottom) has an agent, while the one with the Object handshape (Figure 1, top) does not. The sensitivity of classifier handshape types to such tests is evidence that they are part of the morphosyntax^5^. Besides ASL, adult users of Italian Sign Language (LIS) (Mazzoni, 2009) and NSL also employ this pattern (Goldin-Meadow et al., under review)^6^, as do deaf children acquiring ASL and LIS, but it takes time to develop in children (Brentari et al., 2013, in press).

### Morphophonology in classifier constructions

**Figure 2:**
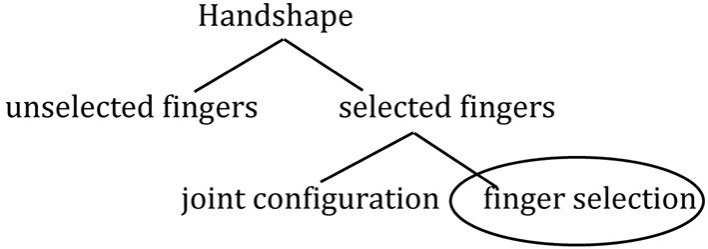
**Position of finger selection (circled) within sign language phonology feature tree for handshape**. Reprinted with kind permission from Springer Science+Business Media B.V.

The hand is not treated as an undifferentiated whole in sign language phonology; handshape has several sub-components. The representation of handshape includes a branch in the feature tree representing the “active,” or *selected fingers* in a given handshape (see Figure 2). Selected fingers are those that move or contact the body during the articulation of a sign. Contrasts in selected fingers constitute minimal pairs and are important for the application of phonological rules in several sign languages, including ASL, ISL, and NGT (Klima and Bellugi, 1979; van der Hulst, 1995; Meir and Sandler, 2007). The features of *selected fingers* form natural classes of handshapes, such as the *index finger group* 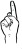, which contains 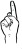, 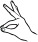, 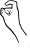, 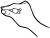, and 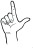; that is, all handshapes with only the index finger selected are a natural class of handshapes, similar to the grouping of obstruents in English^7^.

**Figure 3:**
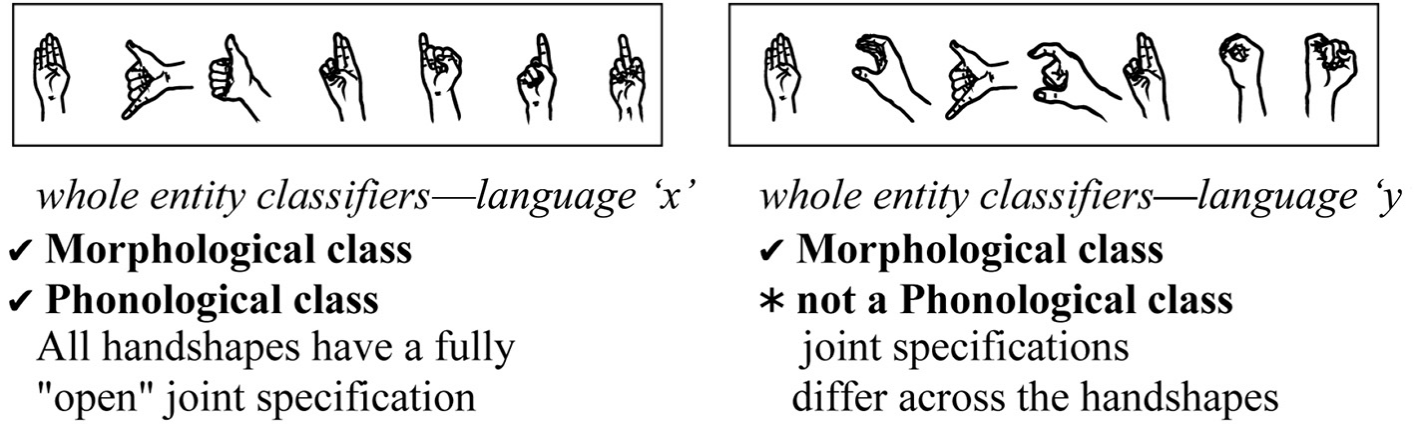
**A group of hypothetical handshapes that would constitute a morphological and phonological class (left) and another group that would constitute only a morphological class (right)**.

The morphological categories for Object and Handling handshapes in classifier constructions might or might not be paralleled by a corresponding phonological pattern. Using joint configuration as an example, let us consider a hypothetical situation in which a given sign language were to use the following set of handshapes as whole entity classifiers—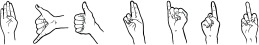. This set would not only be a morphological class, but would also form a phonological class, because the selected fingers in each handshape share a phonological property; namely, they are all *fully open* (“extended”). If a signer of this sign language were to encounter new handshapes, such as 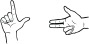 or 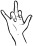, these would be predicted to belong to the whole entity morphological class because of this phonological generalization. In contrast, if a second hypothetical sign language were to use this set of handshapes for whole entity classifiers—
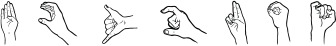
— the set could still be a morphological class, but it would not form a phonological class, as there is no common joint property that the handshapes share. The handshapes would constitute a morphological, but not a phonological, class (see Figure 3); that is, the handshapes in this class would mark a specific class of meanings (whole entities), but without a corresponding phonological property that unifies them. In this second case, a signer could not predict from the handshape structure the morphological class to which it belongs.

Returning to the current study, across a number of sign languages, Object handshapes used in classifier constructions display higher finger group complexity on average than Handling handshapes: ASL and LIS (Brentari et al., 2012), as well as in CSL-S and NSL, two sign languages unrelated to ASL or LIS, and in children acquiring ASL, LIS and NSL after 4–6 years of exposure (Brentari et al., in preparation)^8^. The Object and Handling classifier handshapes in these sign languages therefore exhibit not only morphosyntactic structure but also morphophonological structure of the sort described above—relatively high average finger group complexity associated with Object handshapes and relatively low average finger group complexity associated with Handling handshapes. This distinction in complexity has been described as indirectly iconic: finger group selection is associated with representing the physical properties of objects, and joint configuration associated with manipulating objects, because each aspect is iconically adapted for that phonological task (Brentari et al., in preparation).

### Ranking the finger group complexity of handshapes in sign languages

**Figure 4:**
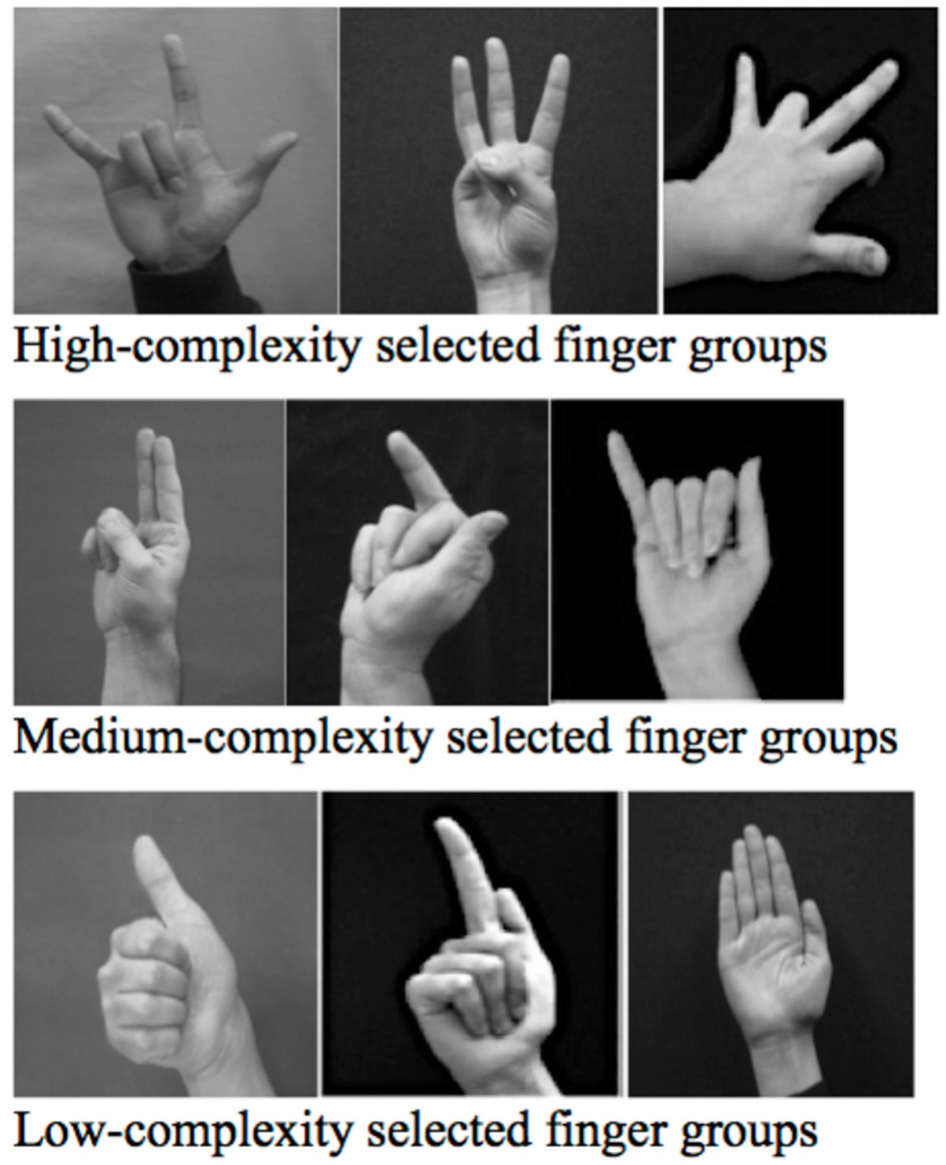
**Examples of handshapes exhibiting selected finger groups of different levels of complexity**. For exposition, in these handshapes the fingers that are fully extended are the selected fingers, and the unselected fingers are the fingers that are fully or partially closed.

Finger group complexity is based on a number of factors, including frequency (Hara, 2003; Eccarius and Brentari, 2008), age acquired (Boyes Braem, 1990), and the number of branches in the phonological structure (Brentari, 1998). Higher finger group complexity also indicates a larger and more mature inventory of handshapes (Marentette and Mayberry, 2000). Handshapes can be divided into three levels of *finger group complexity* based on these criteria. Handshapes with *Low finger group complexity* (Figure 4, bottom) have the simplest phonological representation (Brentari, 1998), are the most frequent cross-linguistically (Hara, 2003; Eccarius and Brentari, 2008), and are the earliest acquired by native signers (Boyes Braem, 1990). They include the groups with all fingers (finger group 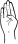), the index finger (finger group 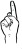), and the thumb (finger group 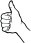). Low-complexity handshapes account for an overwhelming proportion of handshapes in NGT, JSL, and ASL (81% in ASL from Hara, 2003). *Medium finger group complexity* handshapes (Figure 4, middle) include one additional structural elaboration: either a second selected finger on the radial (thumb) side of the hand (the default side), as in finger group 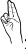, or a single selected digit that is not on the radial side, as in the pinky or middle finger groups 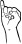 and 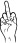. *High finger group complexity* handshapes (Figure 4, top) include all remaining finger groups, which are less frequent and have more complex phonological structures. The correspondence of different levels of finger group complexity to different handshape types is evidence for the two levels of grammar; this organization is not evident in the silent gestures of hearing people (Brentari et al., 2012, in preparation). It is not duality of patterning^9^ because the two levels of structure are not independent, and it is not absolute^10^, but importantly, it reflects organization of the grammatical system at two levels: morphological and phonological.

In summary, iconicity, syntax, morphology, and phonology are complex components of a sign language, and each is acquired along a unique time course. Moreover, the principles of each component interact with one another via interface principles and constraints, as we have seen above: the agent/non-agent distinction (syntax) and the contrast in finger group complexity (phonology) can become manifest only after a distinction between Object and Handling handshapes exists (morphology). Here we examine for the first time the use of Handling and Object handshapes longitudinally in one child homesigner in order to address how such morphosyntactic and morphophonological patterns might emerge.

### How might these patterns arise in homesign?

How might these systematic uses of handshape type and finger group complexity have arisen independently in these unrelated sign languages? Sign languages have their roots in homesign systems, which are gesture systems created by individuals in the absence of a conventional language model (Coppola and Senghas, 2010; Brentari and Coppola, 2012); homesign systems, in turn, use as their raw materials the gestures produced by hearing people in the surrounding culture (Fusellier-Souza, 2006).

A homesigner is a deaf individual whose degree of deafness prevents sufficient access to spoken language to permit acquisition (Goldin-Meadow, 2003). This lack of access to spoken language structure, or to formal instruction, precludes homesigners' learning to read and write. Homesigners also have no or extremely limited access to and interactions with other deaf people, especially to signers of a sign language (either an established sign language, such as ASL for homesigners in the United States, or an emerging sign language, as is used by members of the Deaf community in Nicaragua). Homesigners do not interact regularly with other deaf people, and are not members of a Deaf community^11^.

With regard to the morphosyntactic distinction, adult homesigners as a group behave similarly to users of sign languages and exhibit the agentive/non-agentive distinction (Goldin-Meadow et al., under review). Regarding silent gesture, (Brentari et al., in press) found that hearing gesturers as a group do not use Object and Handling handshape types systematically to express agentivity, and there is considerable between-subject variation; some individual gesturers can produce this pattern (notably adult, Italian gesturers). This contrastive use of handshape is unlikely to be due to the presence of a grammar, as in sign languages. Rather they argue that language, culture, and cognition, as well as the task, contribute to the gesturers' performance. Gesturers are asked to describe minimally contrastive vignettes—they see exactly the same object in the same situation with the minimal difference being the presence of an agent. They are using silent gesture, and therefore channeling all communicative energy into the manual modality. They have a spoken language, and therefore have had a model for the type of meaningful contrast being elicited. Italians also live in a culture that uses a large number of emblematic gestures, which may also provide an additional advantage.

With regard to the morphophonology—higher average finger group complexity in Object-HSs than in Handling-HSs—Brentari et al. (2012) found that in adult homesign systems Object handshape finger group complexity was as high as that of ASL and LIS signers, while homesigners' Handling handshape complexity tended to be higher than signers'. Gesturers do not produce the morphophonological pattern observed in adult and child signers; indeed, as described above, they exhibit large individual differences^12^.

Little is known about the development of these aspects of linguistic structure in children who do not receive conventional linguistic input. In the current study, we will directly compare four adult homesigners in Nicaragua with a child homesigner in Nicaragua, called Julio, performing the same task over time, coded and analyzed in the same way as the adults. Julio stands at the intersection of three different types of populations/participants: (1) as a homesigner, we can compare him to the previously studied adult homesigners; (2) as an individual whose resources for expression in the manual modality are limited to the visual aspects of the language and communication in his environment, we can compare him to hearing, non-signing individuals using silent gesture^13^; and (3) as a child, we can compare him to other children, who are in similar developmental stages, but have different linguistic backgrounds—Deaf children acquiring a sign language from signing parents, and hearing children acquiring spoken language.

### From gesture to grammar: a distributional model

One of the major advantages of our comparative approach is that it allows us to disentangle the contributions of several factors to the emergence and development of linguistic structure: the presence and quality of linguistic input; developmental stage; the function of the gestures/signs in an individual's life (as a primary language for homesigners and signers vs. a one-time occurrence for hearing gesturers); and culture (though this is not directly addressed by the present studies). Because Julio is a child homesigner, we have the unique opportunity to track the changes in his homesign system as it acquires more linguistic structure, from its roots in the non-linguistic gestures produced by the hearing individuals around him. Specifically, we use the distribution of Object and Handling handshapes as a metric of the linguistification of his homesign system^14^. As described previously, this structure is manifested in the distribution of handshapes by exploiting *hand-as-hand* iconicity to use Handling-HSs to express events in which an agent manipulates an object, and exploiting *hand-as-object* iconicity to use Object-HSs for events without an agent. We propose the following model of the emergence of systematic distributions of these two aspects of handshape in the absence of a linguistic model. While these stages are stated in terms of how these aspects of handshape might be selected from the raw materials available in gesture and shaped into linguistic elements, in principle these stages apply to any aspect of form that undergoes the process of transformation from a gesture into a linguistic element *via systematic re-organization and distribution of iconic properties*.

**Stage 1:** Recognizing and using Handshape Type (i.e., Object- and Handling-HSs) as an aspect of form that can be utilized for a meaningful contrast to describe events involving objects and their manipulation. Using the hand in other ways—for example, to trace the path of an object as it moves through space, or as a mere extension of the arm—exemplified by a child who flaps his or her arms to represent an airplane flying—do not reflect this recognition of the affordances of handshape type, and are therefore unlikely to show further development of handshape as a marker of linguistic contrasts. This stage thus represents a potential stage that is not observed when children acquire these grammatical subsystems from linguistic input.

**Stage 2:** Distinguishing between classes of Object and Handling handshapes in one's system; one manifestation of this would be to associate one handshape type (e.g., Object-HSs) with one event type (Non-agentive events), and the other handshape type (e.g., Handling-HSs) with Agentive events. However, this association does not have to be complete in order for these two handshape classes to emerge.

**Stage 3:** Phonological organization that mirrors the morphological organization, but is not necessarily independent from it, i.e., higher finger group complexity in Object-HSs than in Handling-HSs, as shown in Figure 3. Note that difference in the distribution of Object and Handling handshapes is all that is needed before this **morphophonological** pattern can develop.

**Stage 4:** Using Handshape Type to mark a linguistic contrast—i.e., using one handshape type for one purpose and the other handshape type for the other. Specifically, we see the complete association of non-agentive events with Object-HSs (and their on average higher finger group complexity) and agentive events with Handling-HSs, as is observed in the **morphosyntax** of classifier constructions in established sign languages.

Our broad research questions, then, center on how these linguistic uses of handshape develop over time in an individual, in the absence of conventional linguistic input, and their relative timing of emergence. Specifically, in Study 1 we follow the trajectory of the morphosyntactic distinction of handshape type during the 5-year period ending when Julio was about 12½ years old. In Study 2 we follow the trajectory of the morphophonological distinction involving finger group complexity over the same period.

Prior work with child signers suggests that they follow stages 2–4 and may be able to breeze through Stage 1 because they see people signing around them. If Julio behaves like them, his systematic distribution of finger group complexity will precede the sign-like agentive/non-agentive contrast of handshape type (i.e., phonology before morphology). However, if he behaves more like an adult gesturer who is using the resources of iconicity and world experience and who retains as much iconicity as possible in order to better communicate with communication partners who are not skilled users, we would see evidence of the agentive/non-agentive pattern of handshape type earlier than a morphophonological pattern, where the iconicity is less available (i.e., morphology before phonology).

## Study 1: morphosyntax

Supalla (1982) first examined handshape in the acquisition of sign language event descriptions, but did not focus on the agentive/non-agentive opposition. Schick (1987) elicited handling and object classifiers (handshapes) in event descriptions from 24 ASL-learning children (ages 4;5–9;0). Although the children generally used Handling-HSs and Object-HSs correctly, at every age they were more likely to produce correct Object-HSs than Handling-HSs, demonstrating an Object-HS bias (Table 5.3, p. 78). Slobin et al. (2003) found that children learning ASL and NGT spontaneously produced Handling-HSs and Object-HSs as early as age 2;5, but their analysis did not differentiate handshapes used to label objects and actions (e.g., nouns and verbs) from productive classifier predicates (event descriptions, in the terms used here), so it was not possible to determine whether the handshape appeared in a classifier predicate or in a lexical item produced (or created) by the child. Brentari et al. (2013) studied noun/label and classifier/event description Handling-HSs and Object-HSs separately, and found, like Schick (1987), that children produced Handling-HSs for events with an agent less consistently than they produced Object-HSs for events without an agent, and that the agentive/non-agentive opposition is mastered in Deaf children acquiring ASL from native input between 7 and 10 years of age^15^.

Importantly, before handshape can be successfully used to encode a morphosyntactic opposition as described in Stage 1, the child must be producing meaningful (iconic) handshapes. The hands can express events in many other ways, for example, by using an index finger or the whole hand to simply trace the outline or the path of an object that is placed on, or picked up from, the table. In such cases, the movement is iconic but the handshape is not. Other attested examples include using the whole body to represent the movement of an object, such as a child extending her arms to depict an airplane and then leaning over to indicate that the airplane is falling. Both Deaf signing children and child and adult hearing gesturers (Brentari et al., in press), produce such forms, though they are produced much more frequently by non-signers (gesturers asked to respond using only their hands).

We can neither perform traditional syntactic tests that rely on grammaticality judgments (as described in the introduction), nor do we expect minimal pairs or phonological assimilation rules among homesigners and gesturers. However, we have developed methods for identifying such patterns within such systems, if they exist, by comparing the results from such diagnostics with the distribution of handshapes in elicited productions (Brentari et al., 2012). We describe these methods in more detail below.

We focus here on the forms that are comparable to the classifier constructions used by signers; namely, those used in the Event descriptions (i.e., label responses that identified the object were not included)^16^. Here we will ask whether, by the age of 12;8, the child homesigner uses Handling handshapes to express events with agents, and Object handshapes to express events without agents, as in the sign language pattern previously identified. If so, when does this pattern emerge? How does it compare to the patterns produced by adult homesigners, child signers, and child gesturers? To address these questions, we will compare the handshape patterns produced by Julio across the sessions, and then compare his performance with the patterns exhibited by each adult homesigner previously studied in Nicaragua, using the same stimuli, procedure, coding, and analyses. We then ask whether Julio's rate of iconic handshape production (Handling-HSs and Object-HSs together) more closely resembles that of adult homesigners, or that of signing or gesturing children from previously published work (Brentari et al., 2013, in press).

### Methods

#### Participants

***Child homesigner***. The new data reported here were collected from one deaf homesigning child in Nicaragua called “Julio,” who was tested at five time points between the ages of 7;4 (years; months) and 12;8. Julio's family reports that Julio has been deaf since birth. Though audiometry results were not available, his degree of deafness has prevented him from acquiring spoken Spanish. In addition, during the period of the study, Julio did not have sufficient exposure to Nicaraguan Sign Language (NSL) to acquire it. At the final testing session, he began to demonstrate very limited use of NSL signs. The first author became acquainted with Julio through outreach procedures conducted by the Center for Special Education in Estelí, Nicaragua, a medium-sized city about an hour and a half north of the capital, Managua. Julio and his family live in a relatively poor area; his family (all hearing and non-signing) is not very engaged with him, with the exception of his grandmother, who does not gesture extensively with him. Despite repeated visits by first author and the outreach coordinator over the 5-year period of the study to emphasize its importance, Julio's school attendance was sporadic at best. According to his teacher, he did not attend school at all between the ages of 9 and 11^17^.

Because Julio was attending school so sporadically, we observed very little influence of NSL on his homesigns. He acquired very few lexical items, even highly frequent ones: for example, he did not even acquire the NSL count list, a set of signs used often in the classroom, during the period under study. The lack of use of highly frequent lexical items (e.g., *man, woman*) and routine phrases (e.g., *good morning*), combined with our observations of his gesturing with the other deaf children in the classroom who were acquiring NSL, led us to conclude that he was not receiving sufficient exposure to NSL to acquire it during the period of our study. He had no communication partners who used NSL with him outside of the school setting. His brother, who is 2 years older, is his main homesign communication partner; however, recent research examining lexical conventionalization (Richie et al., 2014) and grammatical structure (Carrigan and Coppola, 2012, in preparation) suggests that regular interactions using homesign do not guarantee shared structure between the homesigner and his or her hearing family members.

***Adult homesigners***^18^. Four adult deaf homesigners (1 female) living in Nicaragua also participated in the study (mean age 24, range 20–29 years). The adult homesigners had no congenital cognitive deficits, had not learned spoken or written Spanish, and had not acquired NSL. None had attended school regularly. The adult homesigners did not interact with one another and each had developed a homesign system of his or her own that, unlike Julio, they continued to use as their primary language into adulthood (Coppola and Newport, 2005) and which exhibit a range of linguistic properties, such as pronouns (Coppola and Senghas, 2010) and devices expressing quantity akin to plurals (Coppola et al., 2013). As was the case for the child homesigner for the majority of our study period, the adult homesigners use homesign exclusively to communicate with the hearing people around them, and these hearing individuals often communicate with the homesigner using gestures, though communicative success varies greatly across family groups (Carrigan and Coppola, 2012, in preparation). Each homesigner works, makes money, and interacts socially with hearing friends and family, but is not a member of a Deaf community, and does not have regular NSL communication partners. The first author has worked with three of the adult homesigners since 1996, and the fourth since 2004.

***Child groups***. The analyses in Use of iconic and non-iconic handshape types across groups and Analysis of Specific Handshapes situate the distribution of Julio's handshape types with those produced by child signers and child gesturers (data from Brentari et al., in press). The children in these studies were Deaf native signing children and hearing, gesturing children with no exposure to a sign language responding with silent gestures (3 ASL, 4 LIS, age 3;10–6;4, mean = 5;2); 3 American, and 4 Italian child gesturers (ages 4;3–5;3, mean = 4;8)^19^.

#### Stimuli

**Figure 5:**
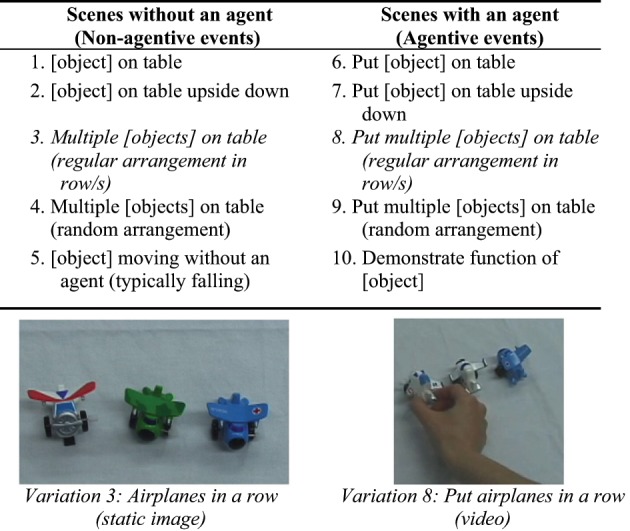
**Descriptions of Non-Agentive and Agentive events, variations in number and arrangement of objects, and specific examples of two stimulus events that contrast only in the presence of an agent**. Reprinted with kind permission from Springer Science+Business Media B.V.

The stimuli consisted of 118 photographs and short videos (henceforth vignettes)^20^. Eleven object types were used in the vignettes: airplane, book, cigar, lollipop, marble, pen, string, tape, television set, and tweezers. The actual objects depicted in the stimulus clips exhibited a range of colors, shapes, and sizes. Each object type was portrayed in 10 variations that fell into two types of events: (1) Five Non-Agentive events, which depicted a stationary object or an object moving on its own without an agent, and (2) Five Agentive events, which depicted an object being moved by the hand of a human agent^21^ (Figure 5). Supplementary Material displays the items presented at each testing session.

#### Procedure

The first author showed each stimulus event to the participant on a laptop computer and elicited a description using minimally verbal instructions: by producing a quizzical facial expression, shrug, and manual flip gesture, often combined with a point. For all sessions, the child homesigner responded to the experimenter (the first author), who has worked with him since he was 6;4, and is very familiar with his gesture system^22^. This procedure successfully elicited gestured descriptions from Julio. The adult participants produced their responses to a family member or friend who was familiar with their homesign: Adult 1 (friend), Adults 2 and 4 (siblings), and Adult 3 (mother).

These descriptions were video recorded, transcoded, and clipped into individual files, one file for each vignette description. The responses were transcribed using ELAN (Crasborn and Sloetjes, 2008; ELAN), a tool developed for multimodal language analysis at the Max Planck Institute for Psycholinguistics in Nijmegen, The Netherlands.

#### Coding

**Figure 6:**
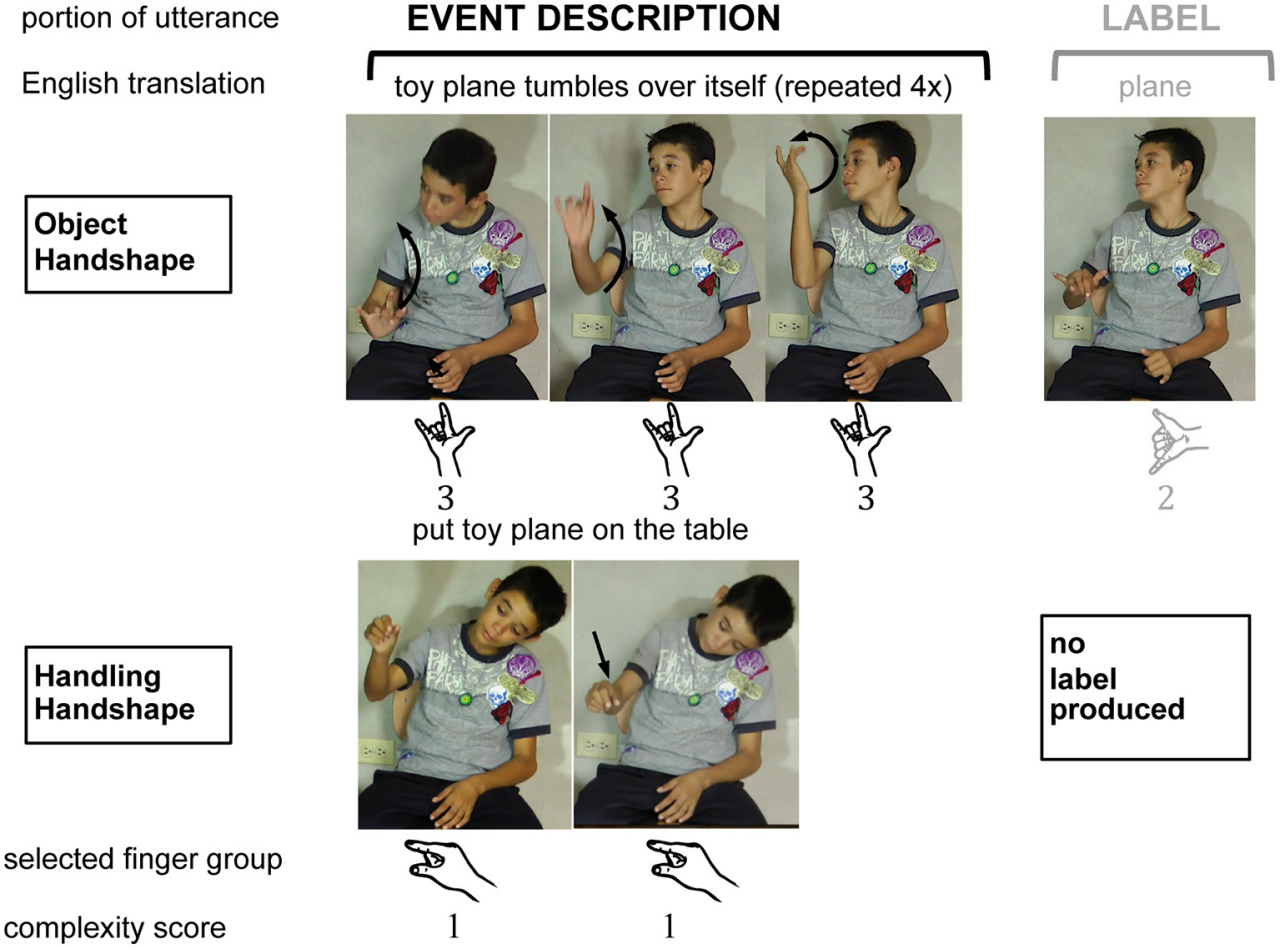
**Examples of two complete responses produced by the child homesigner (for videos, see Coppola, 2014)**. The coding and analysis categories, and the values that were assigned to each form, appear below each photo. All analyses reported in Studies 1 and 2 were conducted only on the signs produced in the Event Description portion of each response. Object handshape (top): Stills taken from a response that was produced to describe the stimulus event *“A mechanical toy airplane tumbles over itself repeatedly.”*; Handling handshape (bottom): Stills taken from a response to the stimulus event *“Someone puts a toy airplane on a table.”* Responses from the adult homesigners were treated identically.

***Coding different components of the response***. We divided the descriptions that Julio produced for each vignette into two portions: *labels* referring to the objects and *descriptions of the event* depicted in each vignette^23^ (see Figure 6). Because all of the vignettes in our study show items on a table or being put on a table, we were able to use a sign's location and orientation to categorize it as an *object label* or *event description*. If the participant produced a form that depicted the movement or arrangement in the vignette, the sign was considered an event description; these were typically produced in a specific location within a single plane, or in relation to a secondary object, most often in the horizontal plane of the signing space (reflecting the fact that the objects in our stimuli were placed on a table). If the participant's gesture was produced on the body or at a nonspecific location in one of the three planes of neutral space^24^, it was considered a label for an object^25^.

***Coding handshape type: Object vs. Handling handshapes***. We categorized each handshape according to type: (1) *Object* handshapes captured properties of the object they represented, either the whole item or size and shape dimensions of the item, and (2) *Handling* handshapes captured properties of the hand manipulating the object. A response was coded as *Both* if: a Handling- and Object-HS simultaneously represented the event on each hand (e.g., a “C” Handling-HS on one hand holding a “B” Object-HS on the other); or the handshape started as a Handling-HS and ended as an Object-HS (e.g., “C” changed to “B”) or vice-versa.

*Both* responses accounted for 6% of the data for events with an Agent and 5% for No-Agent events. In addition, the following handshapes were classified as *Other*: handshapes that “traced” the outline of the object or the path that it took in the vignette (e.g., an index finger or neutral handshape). Handshapes that were neither Handling nor Object handshapes comprised 8% of all productions; 6% for Agent events, and 14% for No-Agent events.

***Reliability***. Two coders transcribed and coded the child homesign productions. They both coded the same subset of 22 items (54 gestures produced overall) to establish reliability of the coding categories. Inter-rater agreement for classifying a given gesture as part of the object label, event description, or other information was 93%; for classification of handshapes according to configuration, 91%, and for classifying handshapes according to type, as *Object, Handling*, or *Other*, 88%^26^. Discrepancies were resolved through discussion.

### Results: agentive/non-agentive distinction using handshape

All of the homesigners produced at least one response per vignette^27^. We first present the results of the longitudinal analysis examining Julio's responses over time (all 11 objects, all conditions, all handshape types), followed by a comparison with the four previously studied adult homesigners in Nicaragua for 4 objects (airplane, book, lollipop, and pen)^28^, followed by a comparison with child signers and gestures on two objects (airplane and lollipop), plus the variation in which an object moved without an agent (e.g., it fell) (all 11 objects).

#### Longitudinal analysis

**Figure 7:**
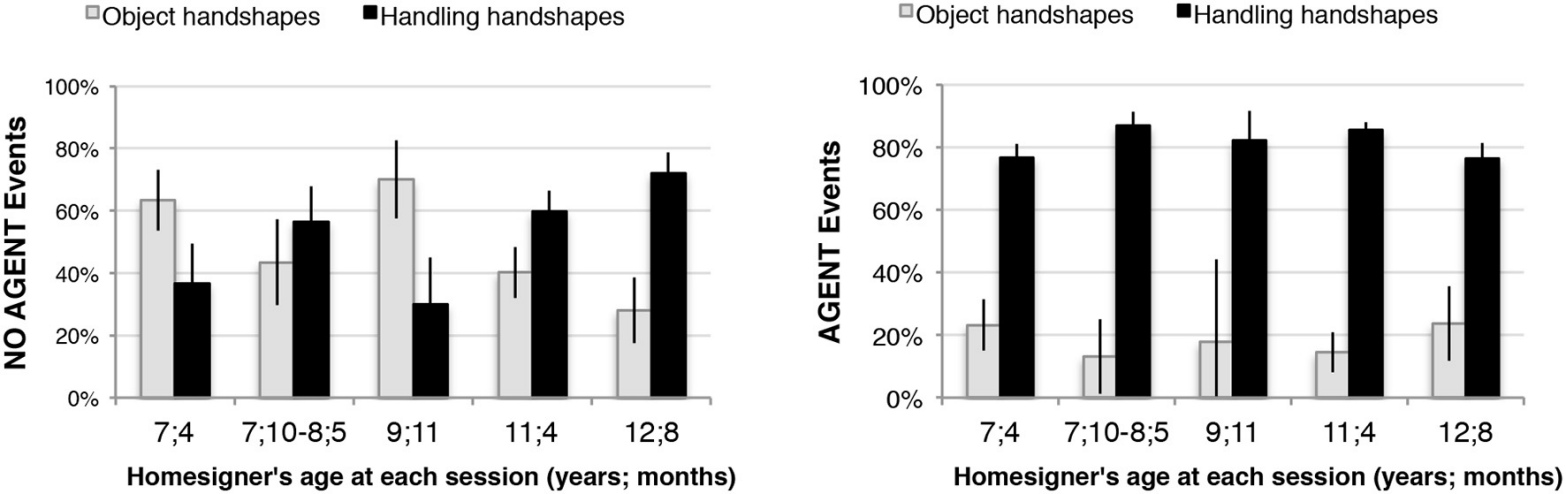
**For events with No Agent, Julio produced the expected Object-HSs on average only 49% of the time overall (gray bars, left chart)**. However, for events with an Agent he patterned more closely with users of established sign languages, producing Handling-HSs in 82% of items overall (black bars, right chart). These analyses are based on responses to all 10 variations from all 11 objects. Error bars indicate 1 standard error.

Julio used handshape contrastively with respect to the presence of an agent from the earliest age studied, although not in the pattern seen in previous studies with adult homesigners or signers of established sign languages. Chi-square tests revealed a significant association between Handshape Type and the presence of an Agent for four of the five testing sessions (Table 1). Across all sessions, Julio, like signers of established sign languages, produced Handling-HSs for events with an Agent (*n* = 258; mean = 82%, black bars, Figure 7, right chart); however, for events with No Agent (*n* = 179), he produced the expected Object-HS on average only 49% of the time (gray bars, Figure 7, left chart). We chose this analytical approach because our primary interest lies in the association between Handshape Type and the presence of an agent (assessed by the Chi-square test), rather than in the relationship between the proportions of Object- and Handling-HSs produced within a particular session. Accordingly, the results in Figure 7 are organized to highlight the different distributions of Handshape Type in No-Agent vs. Agent events^29^.

**Table 1:** **For each session except the last, Julio's responses showed a significant association between handshape type and the presence of an Agent in the vignette**.

**Participant**	**Age**	**Number of responses**	**Pearson Chi-square value (*df* = 1)**	***p*-value two-tailed**	**Phi coefficient**	**Effect size**
Child	7;4	81	13	0.0003^**^	−0.40	Medium
Child	7;10–8;5	95	8.8	0.008^**^	−0.30	Medium
Child	9;11	40	9.72	0.002^**^	−0.49	Medium
Child	11;4	124	10.92	0.001^**^	−0.30	Medium
Child	12;8	97	2.59	0.108		
	Total	437				

** p < 0.01.

#### Julio compared with adult homesigners

**Figure 8:**
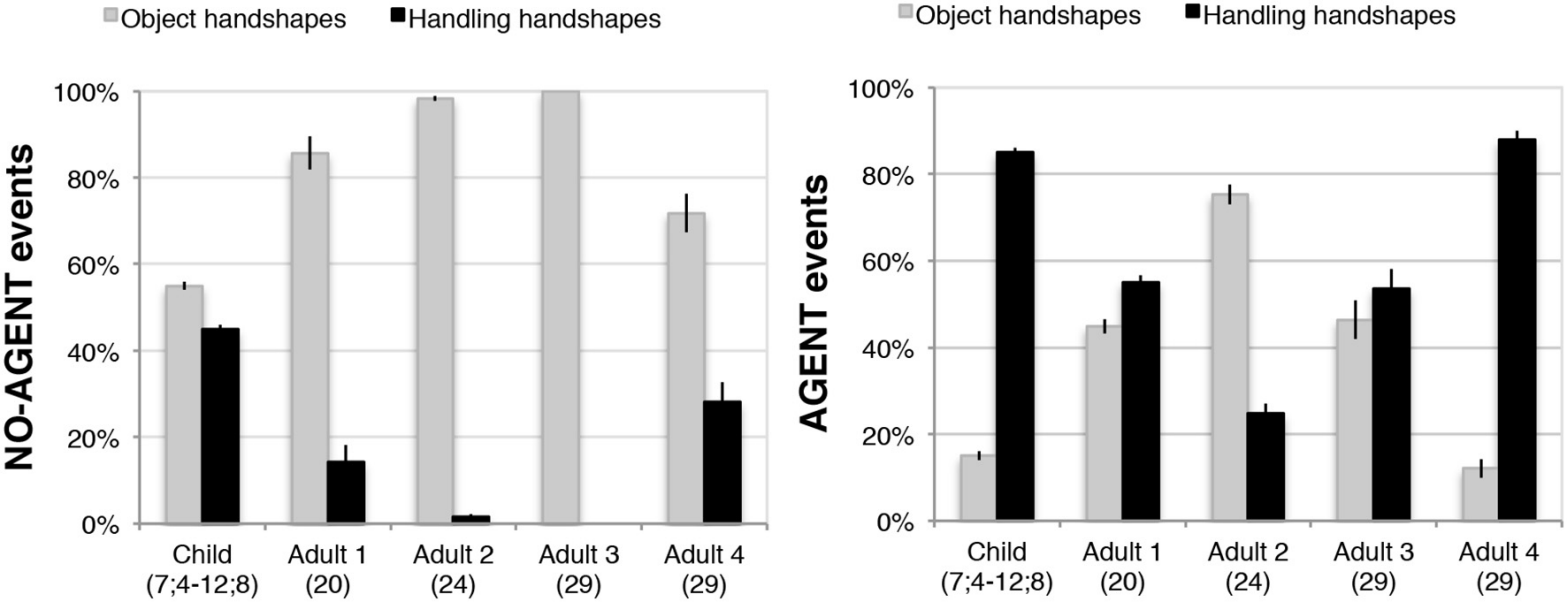
**Comparing Julio with the adult homesigners in Nicaragua in contrastive use of handshape type in events with and without an Agent**. For events without an Agent, all four adult homesigners strongly show the established sign language pattern (a preference for Object-HSs, gray bars), whereas Julio does not (left chart). However, in events with an Agent, Julio does strongly show the established sign language pattern (a preference for Handling-HSs, black bars), as does one of the adult homesigners (Adult 4) (right chart). Data from 4 of the 11 objects are included: airplane, book, lollipop, and pen, because these are the objects for which we have comparable data from the adults. Error bars indicate 1 standard error.

Like Julio, the responses of all four adult homesigners in Nicaragua demonstrated a significant association between handshape type (Object vs. Handling) and the presence/absence of an Agent (Table 2 summarizes the Chi-square analyses). However, Julio's responses to events with an Agent differed from those of the adult homesigners in terms of both pattern type and consistency. Figure 8 shows the proportions of Object-HSs and Handling-HSs produced in each context by Julio and the four adult homesigners. Julio and Adults 1 and 4 preferred Handling-HSs for Agentive events; However, Adult 2 was more likely to produce Object-HSs than Handling-HSs in Agentive contexts, and Adult 3 showed no clear preference. Even more striking differences emerge between Julio and the adult homesigners in their responses to Non-Agentive events. Here, while all four adults were more likely to produce Object-HSs than Handling-HSs, Julio did not show a preference for Object-HSs, as do child and adult signers of established and emerging sign languages.

**Table 2:** **All four adult homesigners and the child homesigner in Nicaragua showed a significant association between handshape type (Handling or Object) and vignette type (Agent or Non-Agent), with effect sizes (a measure of strength of association (Cohen, 1988) ranging from medium to large)**.

**Participant**	**Age**	**Number of responses**	**Pearson Chi-square value (*df* = 1)**	***p*-value two-tailed**	**Phi coefficient**	**Effect size**
Child	7;4–12;8	166	28.84	<0.0001^***^	−0.42	Medium
Adult 1	20	112	34.53	<0.0001^***^	−0.56	Large
Adult 2	24	104	6.88	0.01^*^	−0.26	Medium
Adult 3	29	97	29.67	<0.0001^***^	−0.55	Large
Adult 4	29	89	34.35	<0.0001^***^	−0.62	Large

* p < 0.05,

*** p < 0.001.

#### Use of iconic and non-iconic handshape types across groups

We turn now to a comparison of Julio's responses with those of child gesturers and signers, for context (data previously reported in Brentari et al., in press). While the use of iconic handshapes (i.e., Handling- and Object-HSs, vs. other ways of expressing meaning without using speech) might seem obvious to express events with and without agents, especially when explicitly contrasted as they were in these stimuli, this outcome is not inevitable. Brentari et al. (in press) found that while the child signing groups (in Italy and the US) primarily used iconic handshapes in this task (greater than 90%), the child gesture groups in the no-voice condition used them less frequently (71%). These authors also found a developmental shift in this ability: hearing gesturing children in the US produced fewer iconic handshapes than did American adults. Brentari and colleagues also identified a cultural component to the availability of such iconicity: non-signing adult Italian participants produced iconic handshapes more often than did their American counterparts.

**Figure 9:**
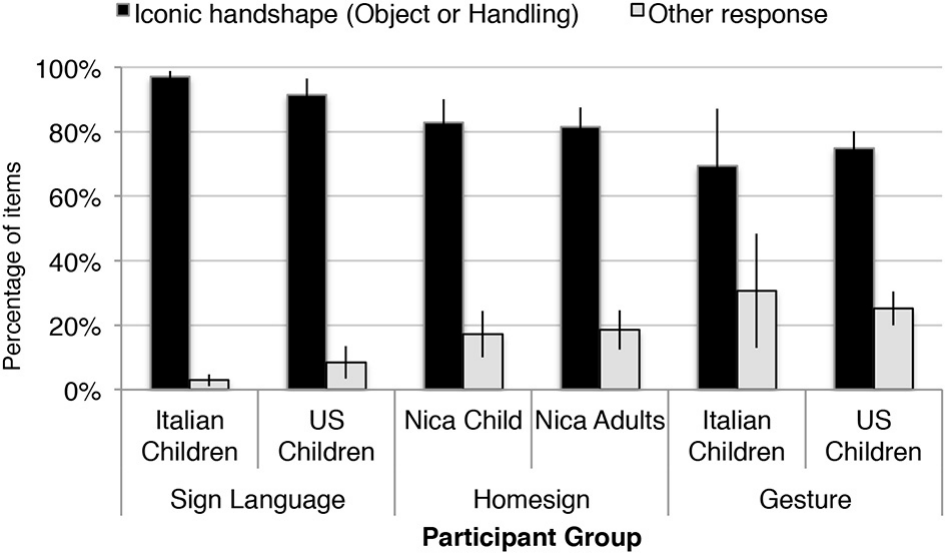
**Similarly to the signing Italian and American children, the Nicaraguan homesigning child and adults tend to produce iconic handshapes to describe the events in the stimulus vignettes**. This analysis includes all stimulus items involving the objects *airplane* and *lollipop*, and all No-Agent trials for all 11 objects in which there was movement (e.g., the object fell). Error bars indicate 1 standard error.

The majority of Julio's event descriptions used iconic handshapes—i.e., Handling, Object or Both. Figure 9 shows the proportion of iconic and non-iconic *Other* handshapes in Julio's responses compared with those of ASL and LIS child signers, homesigning adults in Nicaragua, and American and Italian child gesturers. The rate of producing an *Other* response (e.g., tracing the path of an object with an index finger) was 6% for Agent events, and 14% for No-Agent events (mean 17%), and is similar to that of the adult homesigners (mean 19%). Signing children produces fewer Other handshapes (3% LIS, 8% ASL), and gesturing children produce more (31% Italian; 25% American). Related analyses using data from these participants is reported in Brentari et al. (in press).

### Discussion: use and distribution of iconic and other handshapes

The use of iconic handshapes in sign languages is pervasive; however, it does not necessarily follow that these iconic uses of handshape are immediately accessible in the manual modality to create a system of linguistic contrasts. As described earlier, there are ways to describe the stimulus events that do not make use of either hand-as-hand or hand-as-object iconicity, such as tracing the shape or trajectory of an object. Thus, the first step in developing a system of handshape oppositions that do morphological work is getting into the ballpark by routinely using iconic handshapes to express such events (Stage 1 of our model). The adult homesigners in Nicaragua have used their homesign systems as their primary language over the course of a lifetime. Julio, the child homesigner, compares favorably with them, as well as with child signers, in his ability to use iconic handshapes when responding to these vignettes. Gesturers use more non-iconic handshapes in their responses to these vignettes, suggesting that using the system as a primary language can trigger the prevalent use of iconic handshape to convey meaning even at a relatively young age. Each individual homesign system displays variability, however, in how fully the morphosyntactic opposition is developed.

In Julio the morphosyntactic opposition is not (yet) fully developed. He showed a distributional difference between the use of Object-HSs and Handling-HSs in all sessions; this contrast emerged despite his strong bias to produce Handling-HSs, and it corresponds to Stage 2 of our model. However, his distribution of handshape types with respect to the presence of an agent differed from that of signers, homesigners, or gesturers. The individual systems of the four adult homesigners in Nicaragua showed a significant association between handshape type (Handling or Object) and vignette type (Agent or Non-Agent), with effect sizes ranging from medium to large. But the contrast in the adult homesigners seems largely driven by their propensity to produce Object-HSs in Non-Agentive events (a pattern similar to that shown by children acquiring ASL), whereas the opposition (such as it is) in the child homesigner is driven by his bias to produce Handling-HSs overall. None of the adult homesigners currently shows this bias toward Handling-HSs, but perhaps they did at an earlier timepoint in the development of their gesture systems. How did it arise in Julio? Is a bias toward Handling handshapes a default starting point for homesigners that is later outgrown? Or, if there is a bias, is the handshape type that is initially preferred idiosyncratic, with the opposition building from there? Julio's pervasive use of Handling-HSs to describe vignettes both with and without agents sets him apart from the sign and gesture (pantomime) participants. Julio's pattern is not attested in signers (adults or children), who have a language model, nor is it a pattern seen in adult or child gesturers (Brentari et al., in press). He seems to be working out the system using a different strategy than any of the previously studied groups.

In the larger semiotic context of iconicity, Fay et al. (2013, 2014) have proposed that the greater degree of iconicity afforded by the visuo-gestural modality (vs. the auditory-aural modality) allows faster and more efficient development of human communication systems in the absence of language input. This may be true for human communication, broadly construed, but the present results would suggest that while iconicity is clearly available in the visual realm, its use during the creation of a sign language is much more complicated than its wholesale exploitation. As we see here, the general tendency to use iconic handshapes (of any sort) may be a first indication of relationship between handshape and meaning; however, the specific uses of hand-as-object and hand-as-hand iconicity do not get immediately coopted by the system in a sign language-like way. Somewhat counterintuitively, even though the iconicity of this morphological pattern is quite straightforward, and could be achieved simply by imitating the action of the hand as it is engaged in the action, its development is not easy, quick, or obvious.

In summary, children acquiring ASL do not master this distinction until quite late in language development (Schick, 1987; Brentari et al., 2013); similarly, this seems to be a late developing part of the grammar in an emerging language as well, despite its iconic roots. Julio showed a distributional difference between the use of Object-HSs and Handling-HSs in all sessions, but it was a different pattern than we have seen before, one with a strong Handling-HS bias.

## Study 2: morphophonology

In Study 2 we turn to another level of linguistic analysis, morphophonology, and ask whether Julio, during the time period studied, shows evidence of phonological structure in his homesign system. Previous research using this rubric has demonstrated higher complexity handshapes in Object-HSs than in Handling-HSs representations, as is the case for adult and child users of both established (ASL, LIS, CSL-S) and emerging (NSL) languages (Brentari et al., 2012, in preparation). This pattern has also been observed in adult homesigners in Nicaragua (Brentari et al., 2012). The current study has two goals: (1) identifying when in development this distinction emerges by closely examining the handshapes that Julio produced over a 5-year period in response to targeted vignettes and (2) situating this developmental trajectory in the context of the finger group complexity patterns produced by the same four adult homesigners examined in Study 1.

### Methods: morphophonology

The participants, stimuli, and procedures were the same as Study 1. In addition to the coding procedures outlined in Study 1, we also transcribed the specific handshapes produced using the coding system developed by Eccarius and Brentari (2008), as well as the level of complexity of each handshape. This coding system is based on Brentari's (1998) Prosodic Model of Sign Language Phonology, and was developed using handshape forms from 10 different sign languages. Handshape forms were classified according to selected (i.e., active) fingers and joints. To constrain the number of handshape forms, we did not include non-selected (i.e., inactive) fingers in the criterion for a handshape form. For example, a handshape in which the thumb and index finger formed an “O” would be coded as such whether the three non-selected fingers (the middle, ring, and pink fingers) were curled into the palm or left loosely open. We then categorized forms into complexity groups as described in the Introduction.

Low-complexity handshapes received a score of 1, Medium-complexity handshapes a score of 2, and High-complexity handshapes a score of 3. A small number of gesture/sign responses contained more than one finger group and were assigned the score of the highest complexity handshape contained within it. In these cases, one point was added to the complexity score, regardless of how many handshapes were produced (i.e., two distinct finger groups, or more than two). An example of a gesture containing a handshape change that would not count as a change in finger group (but instead reflects a change in joint configuration) is a C-handshape 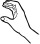 that changes to an S-handshape 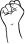 (complexity score of 1). An example of a gesture that contains a handshape change that also exhibits different finger groups would be an F-handshape 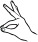 that changes to a stacked B handshape 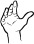 [(total complexity score of 2): both handshapes are low-complexity, so the baseline score is 1, plus 1 for the change in finger group]. On this metric, complexity scores ranged from 1 to 4, where a score of 4 reflected the highest complexity handshapes (value of 3) plus 1 point in the case of a finger group change.

### Results: finger group complexity

#### Longitudinal analysis

**Figure 10:**
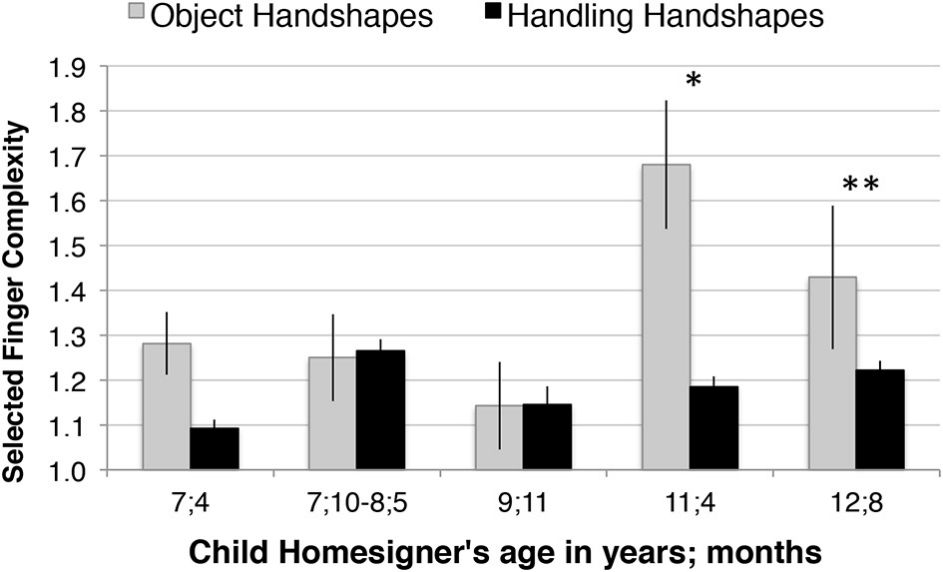
**The established sign language pattern, higher selected finger group complexity in Object-HSs than Handling-HSs, emerges in the child homesigner between the ages of 9;11 and 11;4, and persists**. This chart displays the average selected finger group complexity at each time point. These analyses are based on responses to all 10 variations from all 11 objects. Error bars indicate 1 standard error. ^*^p < 0.05, ^**^p < 0.01.

Figure 10 shows the average finger group complexity for each handshape type (Object, Handling) produced by Julio, the child homesigner, at each session. We first calculated the average finger group complexity for each object (e.g., airplane, book, etc.) within each handshape type, and then averaged across objects. Table 3 summarizes the results of *t*-tests comparing the complexity of Object-HSs and Handling-HSs by session. We found that Object-HSs showed higher average finger group complexity than Handling-HSs only for the last two sessions, when Julio was 11;4 and 12;8. This pattern indicates that the established sign language pattern, higher finger group complexity in Object-HSs than Handling-HSs, emerged in the child homesigner prior to the fourth session and persisted into the next session (12;8). The same pattern was found for handshapes that matched the expected morphosyntactic, sign language pattern described in Study 1 and for “violations” of it (i.e., Handling-HSs in No-Agent contexts and Object-HSs in Agent contexts; see Supplementary Material).

**Table 3:** **Summary of *t*-tests comparing the finger group complexity values for Object- and Handling-HSs produced by the child homesigner at each session**.

**Participant**	**Age**	**Number of responses**	***t*-value**	***df***	***p*-value two-tailed**
Child	7;4	81	1.42	9.91	*ns*
Child	7;10–8;5	94	−0.09	14	*ns*
Child	9;11	40	−0.22	38	*ns*
Child	11;4	124	2.44	37.11	0.02^*^
Child	12;8	97	4.31	95	<0.005^**^
Child	Total	221			

*Non-integer values for degrees of freedom (df) reflect use of the t-test for unequal sample variances*.

* p < 0.05,

** p < 0.01.

**Figure 11:**
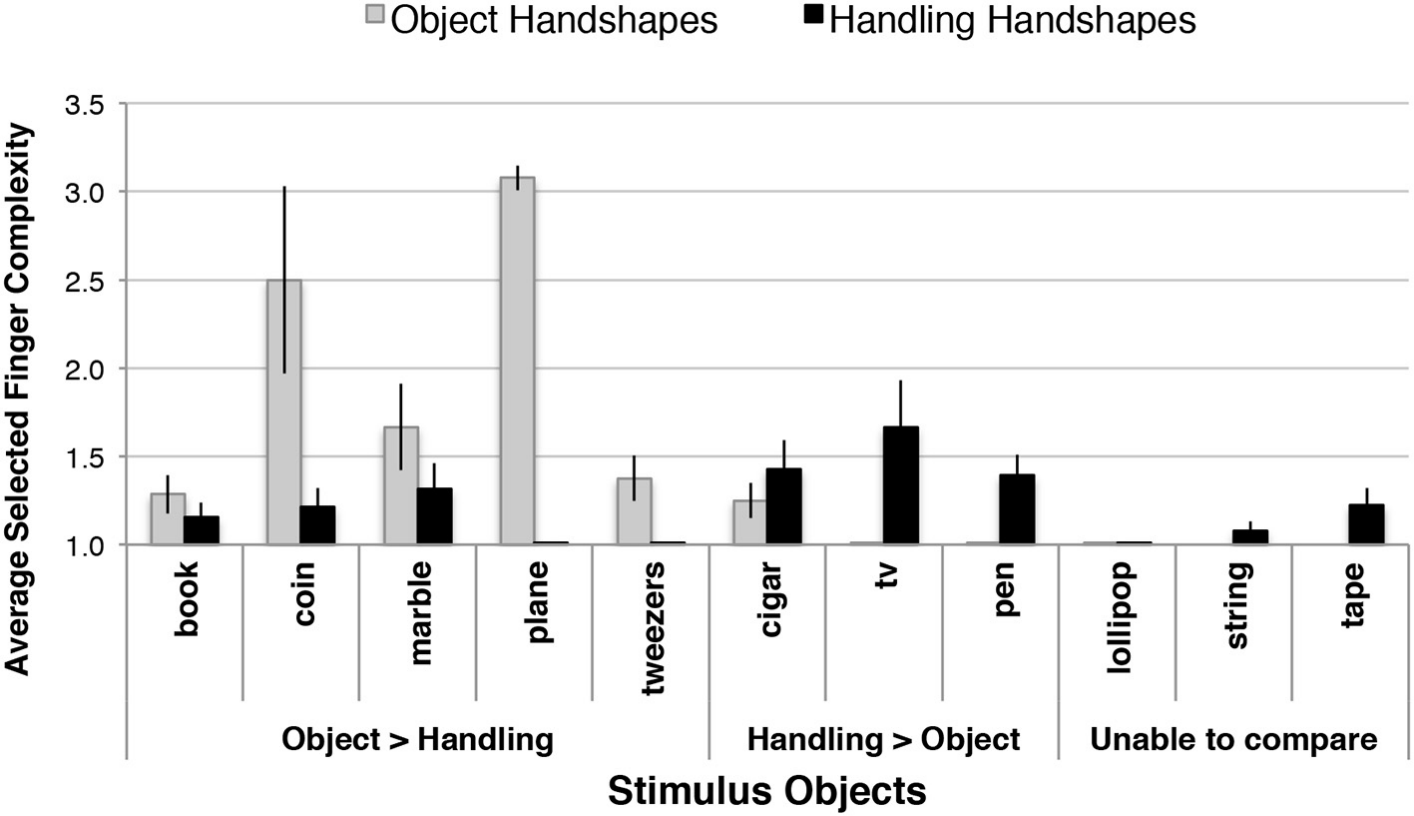
**Selected finger group complexity for the handshapes produced in response to each stimulus object type**. Julio produced Object handshapes with higher complexity for 5 objects and higher complexity in Handling-HSs for 3 objects. Data from all 11 objects, in all variations, were used in this analysis, but comparisons were unable to be made for 3 objects because Julio did not produce both handshape types for these objects. Error bars indicate 1 standard error.

One might wonder whether this higher average finger group complexity in Object-HSs was restricted to a small set of objects (e.g., airplane, given its relatively complex shape). Julio produced between 15 and 26 handshapes for each of the 11 objects studied. Figure 11 displays separately the average finger group complexity for the handshapes produced in response to each stimulus object type. For five objects (book, coin, marble, plane, and tweezers), his Object handshapes exhibited higher complexity, and for three objects (cigar, TV, pen) his Handling-HSs showed higher complexity. He produced only simple handshapes (complexity level of 1) for vignettes featuring lollipops, and no Object-HSs for string and tape, thus we were unable to compare Object-HS and Handling-HS complexity for these three objects. Based on the distribution of finger group complexity across these objects, including those that do not appear to demand high complexity handshapes, we conclude that the morphophonological effect observed in the previous analysis is not isolated to specific objects.

#### Julio compared with adult homesigners

**Figure 12:**
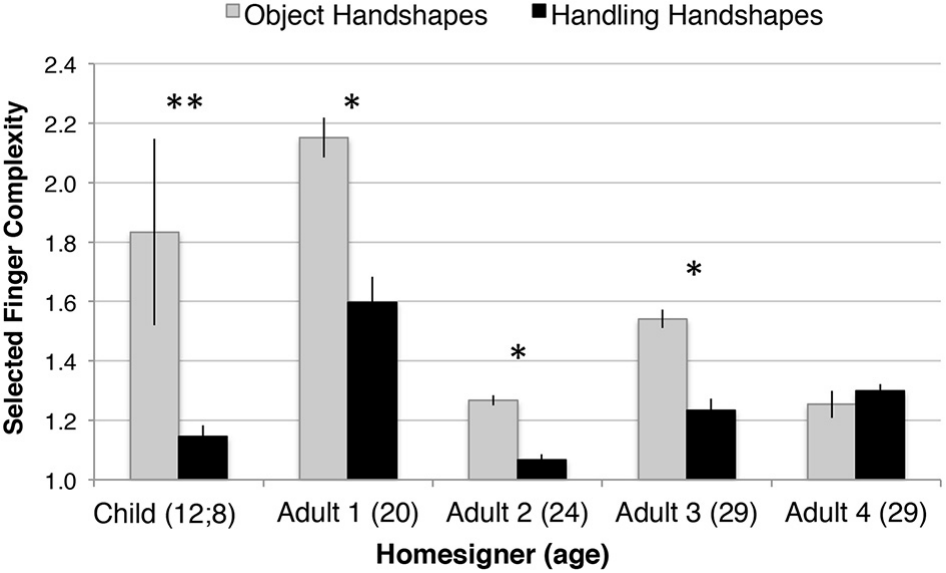
**At the last session tested, the child homesigner (age 12;8) showed the established sign language pattern (higher finger group complexity in Object-HSs than Handling-HSs)**. This pattern was also demonstrated in adulthood by three of the four adult homesigners previously studied in Nicaragua. This analysis includes data from 4 of the 11 study objects: airplane, book, lollipop, and pen, because these are the objects for which we have comparable data from the adult homesigners. Note that a complexity score of 1 is the minimum, and reflects use of the most basic and frequent handshapes observed cross-linguistically. Error bars indicate 1 standard error.^*^p < 0.05, ^**^p < 0.01.

We then compared responses to a subset of four objects (airplane, book, lollipop, and plane) in the child homesigner's last testing session (12;8) (*n* = 97) to those produced by the four adult homesigners previously studied in Nicaragua (total *n* = 402). To standardize the measure of handshape complexity across the different objects so that each object would be weighted equally, we calculated the average finger group complexity across all trials involving each of the four objects described above, and then averaged those, within the sets of Object handshapes and Handling handshapes. Thus, each bar in Figure 12 reflects the average complexity across four objects^30^. For each adult participant, a *t*-test for correlated samples was conducted comparing the complexity of Object- and Handling-HSs produced for each object. Adults 1, 2, and 3 produced significantly higher complexity handshapes for Object- than for Handling-HSs, all one-tailed tests: Adult 1 [*t*_(3)_ = 3.6, *p* = 0.018]; Adult 2 [*t*_(3)_ = 3.12, *p* = 0.026]; and Adult 3 [*t*_(3)_ = 4.53, *p* = 0.010]. For Adults 1, 2, and 3, and for the child homesigner, the mean finger group complexity for Object-HSs was greater than or equal to (in two cases) the complexity of the Handling-HSs produced in response to each object (i.e., book, lollipop, pen, and plane). Adult 4 did not show this pattern: [*t*_(3)_ = −0.16, *p* = 0.442]. In accord with the lack of significant difference found in Adult 4, she showed greater complexity in Handling-HSs than Object-HSs for two objects, lollipop and airplane, equal (low) complexity for book, and only produced handshapes with higher complexity in Object-HSs in response to vignettes featuring pens.

#### Analysis of Specific Handshapes

We now turn to the specific handshapes that were used for each handshape type across participant groups. Figure 13 shows the handshapes classified as Object- or Handling-HSs that were produced in response to events involving airplanes and lollipops (all 10 variations). The child homesigner's data are from the last testing session, after he had begun to show the sign-like finger group pattern. We also provide the same data from the four adult homesigners in Nicaragua, and for the other groups included in the analysis in Use of iconic and non-iconic handshape types across groups: deaf children acquiring ASL and LIS, and hearing children from the United States and Italy (4–6 years of age) who responded using silent gesture. The homesigning and signing groups, but not the gesture groups, produced Object handshapes with Medium- and High-complexity finger groups (Figure 13a). Handshapes with Low-complexity finger groups dominate the Handling-HS responses for all groups (Figure 13b)^31^.

**Figure 13a:**
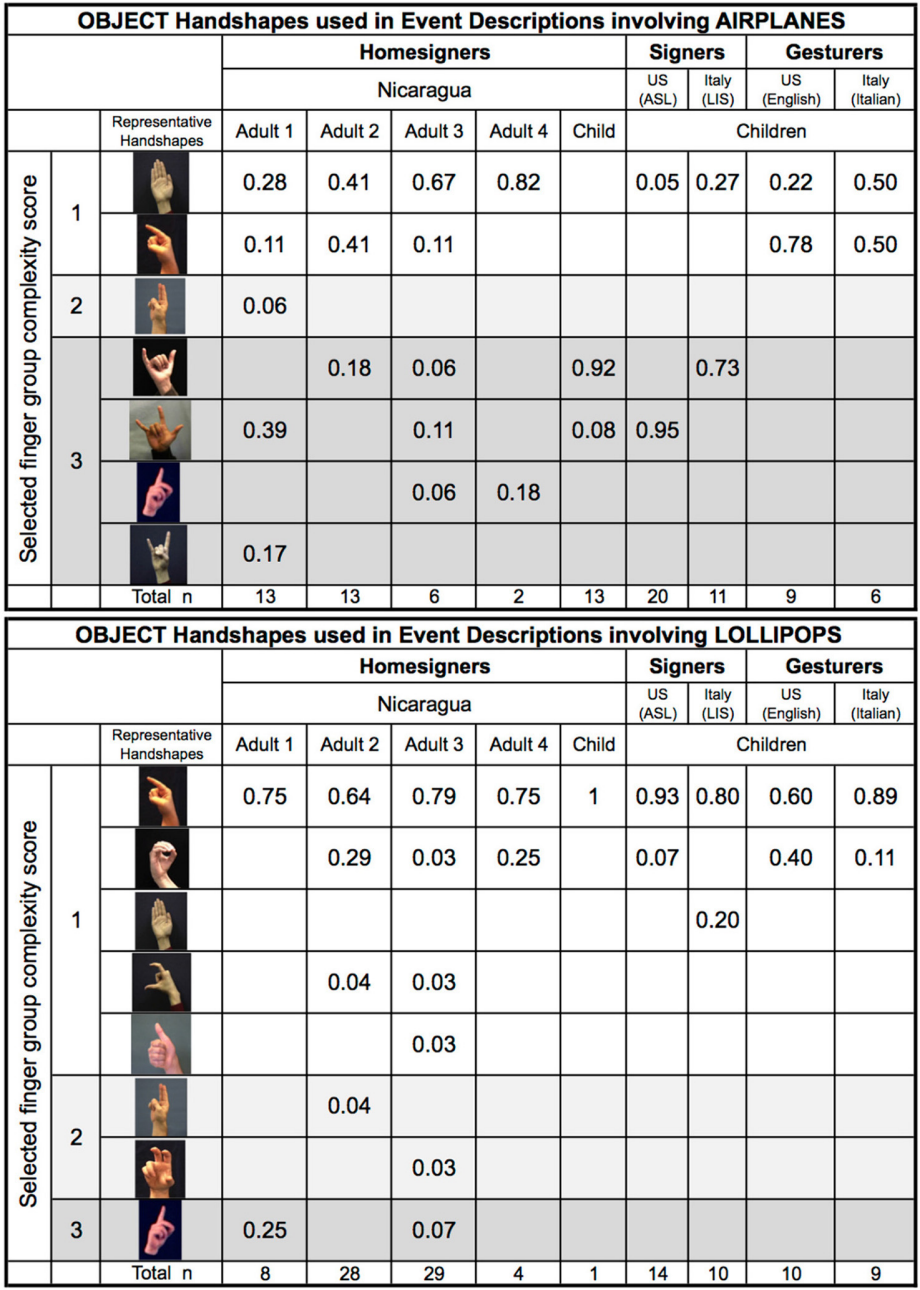
**Object Handshapes used in event descriptions involving Airplanes and Lollipops**.

**Figure 13b:**
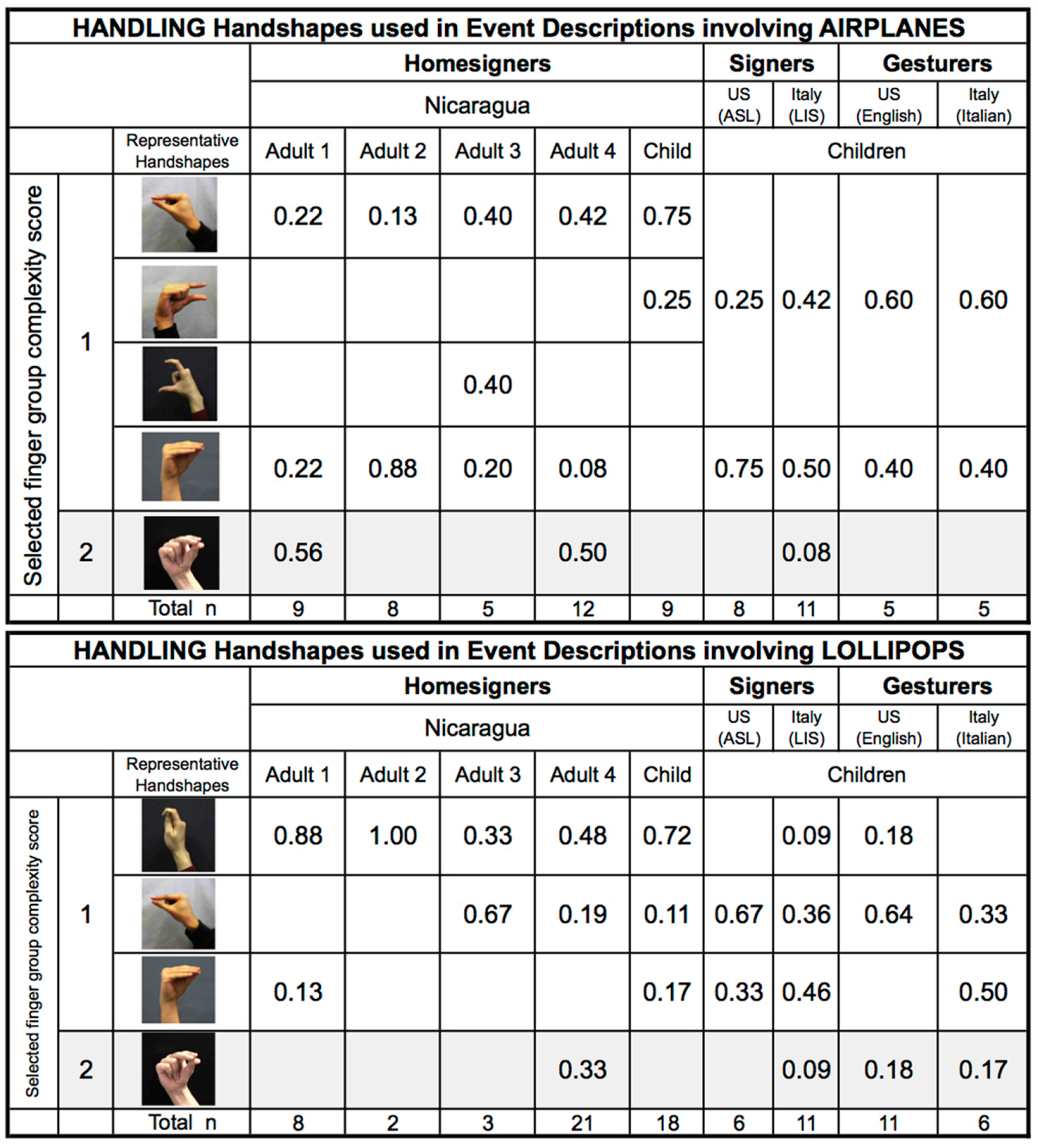
**Handling Handshapes used in event descriptions involving Airplanes and Lollipops**.

### Discussion: morphophonology

We set out to evaluate whether Julio began to show a morphophonological pattern in his use of handshapes during the study period. Specifically, we analyzed Julio's event descriptions for the pattern shown crosslinguistically by adult native signers: higher average finger group complexity in Object-HSs than in Handling-HSs. We found that this morphophonological pattern did emerge in Julio's responses between the ages of 9;11 and 11;4, at which point it was quite robust—it was maintained for at least a year (Figure 10), and it was not restricted to a small number of object types (Figure 11). We also observed higher finger group complexity for Object-HSs than for Handling-HSs in three of the four adult homesigners previously tested in Nicaragua (Figure 12). However, the lack of a linguistic model may affect both the timing and strength of the emergence of this pattern—here, we saw the first evidence of it when Julio was 11;4, several years after we observe its emergence in children acquiring ASL. The relationship between phonological and morphological structure is maintained (phonology before morphology), but the timing is delayed, as has been found in other cases of delayed linguistic input (Morford, 2003; Berk and Lillo-Martin, 2012; Ferjan Ramirez et al., 2012).

Both adult and child signers of established sign languages in the US (ASL) and Italy (LIS) showed this phonological pattern. Some sign languages have relatively short histories and are referred to as “emerging sign languages” (Meir et al., 2010). Brentari et al. (in preparation) asked whether the sign-like distribution of finger group complexity requires multiple generations of signers passing down the sign language. They used the approach previously used with signers of established sign languages with child and adult signers of an emerging sign language, Nicaraguan Sign Language (NSL). They found that, indeed, adult signers of NSL showed the established sign language pattern, as did children with 4–6 years of exposure. Thus, this pattern emerges relatively early in development when children are acquiring a sign language from linguistic input^32^.

None of the hearing adult or child gesturers we have tested, in the US, Italy, or Nicaragua, showed this. Gesturers show very little finger group complexity at all, or else the opposite pattern of higher complexity in Handling-HSs, rather than in Object-HSs (Brentari et al., 2012, in preparation). The high- and medium-complexity finger groups observed in signers' Object-HSs (such as handshapes in which the index and middle fingers are active) were rarely used for Handling-HSs (Eccarius, 2008; Brentari and Eccarius, 2010), even though these handshapes are used in daily life to manipulate certain objects (e.g., holding a baseball or grasping a small teacup by its handle).

In summary, these results, combined with the present results from Julio, suggest that the morphophonological pattern does not appear to require linguistic input in order to emerge, and that it is not inevitable when using the manual modality.

## Overall discussion

We began by identifying universals in the ways that sign languages use two aspects of handshape: (1) handshape type and (2) finger group complexity, to mark linguistic contrasts. Importantly, in sign languages handshape type—the systematic use of Object-HSs vs. Handling-HSs, depending on the presence of an Agent—is grammatical: the distribution of handshape types is associated with a meaning contrast (Agentivity) and thus constitutes a morphosyntactic system. These handshape types are also systematically associated with contrasting levels of average finger group complexity—higher in Object-HSs than in Handling-HSs—and are in this sense morpho-phonological. Recall that the phonological patterns we observed are embedded within the morphosyntactic structure of the classifier constructions of sign languages. We discovered a degree of convergence between these language universals for sign languages that have classifier systems, and in the behavior of individual child and adult homesigners. We then attempted to identify the source of this convergence—specifically, whether it could be attributed to shared constraints on iconicity. We turn now to an integrative discussion of how the distributions of handshape type (Object vs. Handling-HSs) and finger group complexity that we observed in the child homesigner Julio constitute linguistic, and specifically, morphophonological development, rather than mere elaborations of iconic patterns that emerge from the affordances of how humans interact with objects. Specifically, we show how the results and analyses from Studies 1 and 2 serve to evaluate our proposed model of the emergence of systematic distributions of handshape type and finger group complexity in the absence of conventional linguistic input.

Returning to the *Distributional Model* proposed in the Introduction, we can now insert our findings from the two studies presented:

**Stage 1:** Recognizing Handshape Type as an aspect of handshape form that can be utilized for grammatical purposes.

*Finding:* Julio predominantly uses Object and Handling (iconic) handshapes, rather than neutral handshapes or full-body expressions, to describe events involving objects and manipulation (see Figure 9). Rooted in *hand-as-object* and *hand-as-hand* iconicity, they can be thought of as the raw materials from which a morphological system can be constructed.

**Stage 2:** Distinguishing the distribution of Object and Handling handshapes in one's system; associating one handshape type with one event type and the other handshape type to the other event type *to some degree*. This association does not have to be complete, nor does it have to be present for both handshape types/event types, in order for a contrast to emerge between these handshape classes.

*Finding:* Julio makes this distinction, manifested by his different distributions of handshape type to express events with and without an Agent (see Table 1 and Figure 7). We interpret his association of Handling handshapes with Agentive events, and to a lesser degree his association of Object handshapes with Non-agentive events as evidence of this. These two handshape classes thus lay the foundation for the phonological pattern to appear.

**Stage 3:** Organizing phonological properties with regard to handshape classes. This organization need not be independent from the morphological category to be phonological (see Figure 3). Note that a contrast between agentive and non-agentive events that is encoded in handshape is all that is needed before this **morphophonological** pattern can develop.

*Finding:* By the second-to-last session, Julio's Object-HSs displayed higher average finger group complexity than his Handling-HSs, and this pattern was not restricted to a small number of objects (see Table 3 and Figures 10, 11).

**Stage 4:** Using Handshape Type systematically and oppositionally to mark the presence or absence of an agent, as is observed in the **morphosyntax** of classifier constructions in established sign languages^33^.

*Finding:* In contrast to the robustness of Julio's homesign system with respect to Stages 1 through 3 of the model, he does not show evidence of using handshape to mark this grammatical opposition (see Table 1 and Figure 7); nor do two of the four adult homesigners whose handshapes have been studied using the same stimuli and analytic procedures (see Table 2 and Figure 8).

Interestingly, the adult homesigners show a range of outcomes with respect to the model. Like Julio at the end of the study period, Adults 2 and 3 have reached Stage 3 (showing the morphophonological pattern, but not the full morphosyntactic opposition). Adult 1 has reached Stage 4 (showing the morphophonological and the full morphosyntactic opposition found in sign languages). Adult 4's performance is atypical: though she shows the morphosyntactic opposition of Stage 4, she did not develop the morphophonological pattern (Stage 3). One explanation might be that she did not develop a sufficiently large or complex inventory of handshapes that could then be used differentially in Object and Handling handshapes. We leave this for future work.

Within this productive subsystem of a sign language, the morphophonological pattern develops earlier than the morphosyntactic one in child signers as well as in this single Nicaraguan child homesigner whose exposure to NSL was extremely sparse and sporadic. The results of the two studies, and their relation to the proposed model, are summarized in Table 4.

**Table 4:** **Brief descriptions of the stages of the Distributional Model and summary of results from Studies 1 and 2**.

		**Users of established sign languages**	**Homesigning participants**					
			**Child 7;4**	**Child 11;4**	**Adult 1 20 years**	**Adult 2 24 years**	**Adult 3 29 years**	**Adult 4 29 years**
Predominant use of iconic handshape types (Object- and Handling-HSs		Yes	Yes	Yes	Yes	Yes	Yes	Yes
Morpho-syntax	Association between handshape and agentivity (Stage 2)	Yes	Yes	Yes	Yes	Yes	Yes	Yes
	Agentive events	Handling-HSs	Handling-HSs	Handling-HSs	Handling-HSs	Object-HSs	No strong preference	Handling-HSs
	Non-agentive events	Object-HSs	No strong preference	No strong preference	Object-HSs	Object-HSs	Object-HSs	Object-HSs
Morpho-phonology	Greater complexity for Object-HSs than Handling-HSs (Stage 3)	Yes	No	Yes	Yes	Yes	Yes	No
Systematic opposition of handshape type between agentive and non-agentive events (Stage 4)		Yes	No	No	Yes	No	No	Yes

One can ask whether Julio would behave more like a child signer in his development (phonology before morphosyntax) or more like some adult gesturers who produce the target handshape distinction based on the presence of an agent without a corresponding phonological level of structure. Julio apparently behaves more like a child signer than an astute Italian gesturer: he produces the phonological pattern in finger group, but not the expected pattern in handshape type. An important point regarding comparing homesigners and gesturers on the same task is that homesigners come to the task with experience of using their system on a daily basis to express a variety of meanings and grammatical contrasts, while the gesturers are inventing their responses on the spot. The homesigners, therefore, are constantly balancing and integrating multiple aspects of their systems, while the gesturers are presented with a single, specific communicative task that has been tailored into a bite-sized chunk. Differences between Julio and adult Italian gesturers exemplify the consequences of moving beyond this restricted domain of solving a single communication problem by expressing Agentive vs. Non-Agentive events, to the complexity of trying to solve the multi-dimensional problem of expressing a variety of contrasts simultaneously in the creation of a linguistically organized system. The latter is the task faced by a single homesigner in the absence of linguistic input.

### Phonological development and morphology in signed and spoken languages

Previous studies of the acquisition of phonology and handshape in sign languages have focused almost exclusively on the timing and patterns of acquisition of lexical nouns and verbs, or on the acquisition of semantic classifier forms, such as Object/Entity classifiers. However, most studies have converged on a common conclusion, that different formational parameters tend to be acquired in a piecemeal fashion (i.e., different timing for the acquisition of location and handshape configuration; see, for example, Boyes Braem, 1990; Marentette and Mayberry, 2000; Meier, 2006; Ortega and Morgan, 2010). Further, the core lexicon is not the only place within a grammatical system that one might look for evidence of phonological structure (as in, for example, Kantor, 1980; Fish et al., 2003; Eccarius, 2008)—our study uniquely addresses phonological patterns that take into account the morphosyntactic function of the classifier construction, and exist beyond the domain of the lexicon. Considering phonological structure more broadly, then, our observations of Julio indicate that phonology appears relatively early, in the form of contrastively used finger group complexity.

The relatively late acquisition of morphosyntactic patterns in ASL and other sign languages, and the close interplay between phonology and morphology, is not unique to sign languages, but is also found in spoken languages (e.g., MacWhinney, 1978; Levinger-Gottlieb, 2007; Ravid and Schiff, 2009). Since previous work demonstrated that three of four homesigners tested in adulthood already showed the morphophonological pattern that Julio displayed here in later sessions, we can also now put this finding into the broader context of phonological development in the absence of a linguistic model. The present work represents the first study of phonological and morphosyntactic development in the use of handshape over time in a homesigner of any age who has yet to be immersed in a sign language environment. It also adds to a very small literature addressing the development over time of any linguistic structure in homesign systems that continue to be used as primary languages beyond early childhood^34^.

### Iconicity and morphosyntax

If hand-as-hand and hand-as-object types of iconicity are widely accessible to all populations, we might expect anyone who responded to these vignettes to show the morphosyntactic*-like* opposition described in Stage 4 100% of the time. Indeed, the participants across all language groups and ages in these studies could have achieved the sign-like morphosyntactic pattern by simply mimicking the actions of the human agent in the Agentive events. Likewise, they could have succeeded in the No-Agent events by refraining from inserting an Agent into the event, that is, by using any non-Handling handshape to express the arrangement or movement of the object(s) in the vignettes (e.g., a simple handshape such as, 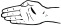). But this is not the pattern we have observed. Julio failed to exploit fully and equally the iconicity present in Handling- and Object-HSs. One possibility is that he did not grasp the distinction between Agentive vs. Non-Agentive events. While we cannot rule this out, Julio certainly appeared to understand the task; the instructions given to all participant groups are quite minimal, namely “describe what you see.” His strikingly different handshape distributions across the two types of events suggests that he was sensitive to the presence of an agent^35^. Moreover, we have seen this pattern across a number of other populations: in the adult homesigners in Nicaragua also reported here; among Cohort 1 and 2 signers of NSL and native signers of ASL (Goldin-Meadow et al., under review), as well as among children acquiring ASL and LIS (Brentari et al., in press).

Brentari et al. (in press) argue that while cognitive and cultural factors influence the use of the handshape for this purpose, there is also a strong linguistic component to this opposition, as argued in Benedicto and Brentari (2004); thus handshape used in a systematic, motivated way to mark agency and transitivity, as described above, is *both* a specifically motivated iconic pattern (one that even some gesturers can discern), but used in the service of grammar in sign languages (cf. Meir et al., 2013).

The argument that Julio only “overuses” hand-as-hand iconicity, on our view, constitutes evidence for different subtypes of iconicity and is the foundation for our claim that Julio's system is moving beyond what is offered by perceptual/cognitive affordances and into a linguistic realm. This linguistification is driven by the continued need to develop the system itself (in the absence of a linguistic model), and the consequent requirement that forms exist in relationship to other forms, rather than just being associated with meanings in the world. Converging evidence comes from other studies of homesign systems developed by children in the US and China, which exhibit morphological structure, i.e., handshape categories and motion categories that combine productively to create new signs (e.g., Goldin-Meadow et al., 1995, 2007). It is more appropriate to characterize Julio's bias toward Handling-HSs as an incomplete re-organization of iconicity by his grammar. Julio uses as many iconic handshapes as signers do at the same age. Moreover, the predicted sign language pattern is based on iconicity as well (the two types: hand-as-hand and hand-as-object), and Julio shows a different distribution of his use of iconicity.

### Iconicity and morphophonology

Like the morphosyntactic pattern, the morphophonological pattern is also iconic, though in a different, more indirect way, and with a less direct relationship between the stimuli that the participants saw and the task they were asked to perform. If there were an obvious solution to expressing that iconicity, presumably gesturers (certainly adults) would also demonstrate it, but the evidence from gesturers in three different cultures suggests that they do not (Brentari et al., 2012, in preparation). Our study specifically investigated the use of handshape to mark this opposition. However, it is possible to imagine other parameters of sign formation, such as movement, representing the first step toward marking the agentive/non-agentive distinction. One form this might take would be to use “contact” movements (i.e., movements with a final “bump” that highlight the “surface” on which an object rests) to express stative events vs. events with movement, which would overlap some with the agentive/agentive distinction, but not completely (e.g. objects that fall). We leave this for future work.

Several researchers have argued for a crucial role of iconicity in the development of structure in the manual modality, in both emerging systems such as homesign as well as in established sign languages (see, for example, Cuxac's (1999) work on iconicity from a semiotic perspective in French Sign Language). It has been argued that users of homesign must rely on iconicity in order to maintain transparency and comprehensibility^36^ (e.g., Goldin-Meadow, 2003; Fusellier-Souza, 2006). In the domain of experimental semiotics, Fay et al. (2013, 2014) have argued that iconicity can facilitate human communication in the absence of linguistic input. This use of iconicity is pre-Stage 1 in in our model, because different ad hoc strategies for employing different kinds of iconicity might suffice for a single, relatively constrained communicative task, but not for a primary language that has to serve many functions. And while iconicity facilitates communication, the present results suggest that iconicity is not sufficient to build a linguistic system.

### Language emergence and evolution

The researchers studying another recently emerged language, Al-Sayyid Bedouin Sign Language (ABSL) have observed that morphology appears to be developing more quickly than phonological structure (e.g., Sandler et al., 2005; Padden et al., 2010; Sandler et al., 2011). The morphological structure they have studied relates to verb (person) agreement, which appears in signers of the first generation of ABSL. The present results from Julio, a child homesigner studied longitudinally, seem to indicate the opposite: that phonological structure can appear relatively early, while morphological development requires more time. We can think of at least three ways to reconcile these apparently contradictory findings: (1) Morphology and phonology are expressed in different ways in different subsystems of the grammar. Our study focused on the use of handshape to mark contrasts in agentivity, while the morphological aspects of verb/person agreement involve the movement of verb signs in signing space. Further, examinations of phonology in the context of morphology, comparable to those under study here, have not been reported in ABSL. (2) Perhaps the type of structure they put forward as morphological, reflecting the notion of “body-as-subject” and involving movements of verbs along the midsagittal plane of the body, are anaphoric at the level of discourse rather than at the morphosyntactic level. These authors also suggest this as a possible explanation of their findings. (3) ABSL is a village sign language, in which deaf and hearing users interact with each other, and many deaf individuals use the language as their primary language. This is a different sociolinguistic setting from that of homesign, the focus of the current study. Julio and the adult homesigners in Nicaragua do not experience the same pressures to conventionalize their linguistic systems with the hearing people around them, who do not use the system as a primary language. Without this pressure, perhaps phonological complexity has a higher probability of emerging. It may be that regular interactions with individuals who do not use the manual system as their primary language (i.e., hearing communication partners) hinder the homesigner's internal consistency (see Richie et al., 2014; Goldin-Meadow et al., under review, on the conventionalization of lexical items in the individual homesign family groups).

Advantages of our comparative approach include the ability to separately identify the contributions of several factors to the emergence of linguistic structure: linguistic input; stage of development; use of the manual modality as a primary language for homesigners and signers vs. a one-time occurrence for hearing gesturers; and culture. To address the role of repeated and habitual use of the manual modality to express ideas/concepts that would typically be expressed in speech in hearing individuals, in ongoing work we are following hearing, non-signing gesturers who do not have regular contact with homesigners, as well as homesigners' regular communication partners over time.

Homesign systems differ in significant ways from sign languages used by a community of Deaf people (e.g., Spaepen et al., 2011, 2013 on the lack of a count list; and Richie et al., 2014 and Goldin-Meadow et al., under review on the slower conventionalization of lexical items). However, the available evidence strongly indicates that homesign more closely resembles sign language than it does gesture. This evidence, unsurprisingly, comes from studies of linguistic structures that do not involve conventionalization among a community of users (e.g., Coppola and Senghas, 2010 on pronouns; Brentari et al., 2012 on morphophonology; Coppola et al., 2013 on plurals). In accord with the findings summarized above, careful consideration of the relationships among the individual structural components exhibited in homesign reveal that constraints across different levels of linguistic analysis are much weaker than they are in either emerging or established languages, which have the benefit of a linguistic community and/or linguistic input. When linguistic input is available, it apparently constrains multiple levels of linguistic structure simultaneously, but without linguistic input, cohesion and integration across components of the grammar is less apparent, and we can see the piecemeal development of sub-parts of both morpho-syntax and morpho-phonology.

## Conclusion

Perhaps because of the affordances offered by a language system using the manual modality, the notion of iconicity as a highly complex, multi-layered set of phenomena that are utilized in distinctly different ways in sign languages is often not fully appreciated. Brentari (2007) notes that while it is clear that iconic sources can be identified for many aspects of sign language structure, it is also evident that “arbitrary formal structure is present and observable at every level of SL grammar.” Indeed, this notion is echoed in the present findings from one child homesigner, in which we observe a less iconic form (contrastive use of finger group complexity) emerging earlier in development than a type of iconicity that appears more straightforward (namely, hand-as-hand iconicity). We interpret his lack of exploiting the hand-as-object iconicity as a consequence of the fact that, as a homesigner, he is building a grammatical system from non-linguistic gestural input.

These findings constitute evidence that individual components of a phonological system can exist before there is a full phonological system (e.g., one that includes minimal pairs and assimilation rules). In this regard, these results accord with previous findings with adult homesigners (Brentari et al., 2012) and children acquiring an established sign language (Brentari et al., 2013). Some adult gesturers achieve Stage 1 of our Distributional Model, where handshape is meaningfully manipulated (Brentari et al., in press) and signing children and adult homesigners achieve all four stages of the model, where the opposition of handshape in phonology and morphology is clear (Brentari et al., 2012, 2013, under review). The evidence we have described here shows that a single child homesigner has achieved Stage 3 (morpho)-phonology), but not Stage 4 (morphosyntax). The particular manifestation of these components in both child and adult homesign systems, while not deterministic, nevertheless generally accords with the associations seen in emerging and established sign languages. In other words, while a homesigner will not achieve the same level of linguistic sophistication, in both homesign and sign languages, iconicity is dismantled and reassembled in the service of a multi-componential system. Further, these distributional patterns do not reflect the patterns observed in the gestures produced by hearing people to describe these vignettes in the manual modality, who may astutely exploit available patterns of iconicity and their life experience with co-speech gesture and a spoken language and apply these skills to a specific gestural “problem” presented in a controlled task.

While it is difficult to generalize from a set of five case studies, we take the child and adult homesign findings as an existence proof that some aspects of morphophonology and morphosyntax can develop within an individual who is not acquiring a conventional language. Considered in conjunction with related studies, these findings also suggest that iconicity, in the sense of the hand representing the hand of an agent, or representing an object's movement or properties, does not entirely drive these linguistic developments. Nor is this iconicity easily accessible to individuals gesturing without voice (pantomime) who do not routinely communicate in this fashion. Ongoing analyses of the gesture descriptions produced by the communication partners of the child and adult homesigners offer an opportunity to distinguish these factors. Taken together, this body of work suggests that, while handling and object handshapes are ubiquitous and iconic, the various cognitive and linguistic roles these handshapes can assume cannot be conflated and must be investigated independently, and more importantly, analyzed distributionally.

### Conflict of interest statement

The authors declare that the research was conducted in the absence of any commercial or financial relationships that could be construed as a potential conflict of interest.

## Footnotes

^1^For convenience, all of the sign languages mentioned in this paper and their abbreviations are given here: Al-Sayyid Bedouin Sign Language (ABSL), American Sign Language (ASL), Chinese Sign Language (Shanghai dialect; CSL-S), Italian Sign Language (LIS), Israeli Sign Language (ISL), Japanese Sign Language (JSL), Nicaraguan Sign Language (NSL), and Sign Language of the Netherlands (NGT).

^2^We will refer to this as “finger group complexity” henceforth.

^3^As one reviewer suggested, it is an open question whether a system will express one meaning with both handshape and movement fused together or whether handshape and movement will have componential meanings. Evaluating this hypothesis will require a parallel and systematic analysis of movement, similar to the one for handshape presented here. This is beyond the scope of this paper, but is an active area of our current work.

^4^To be as neutral as possible about the linguistic status of such gestures in the different groups we will be comparing, we refer to these as “labels.” Iconic handshapes have a different distribution in labels/nouns, and because we are comparing the portions of participants' responses that are most comparable to sign language classifier constructions, we exclude the labels from the analyses in the current study.

^5^This phenomenon is phrasal because it is the combination of lexical semantic features that causes the ungrammaticality. So it can be analyzed as either phrasal semantics, or phrasal syntax. Under a syntactic model that allows the features of the lexical semantics to be fed into the syntactic tree for feature checking (e.g., Borer, 2005) it is considered part of syntax.

^6^While many studies have investigated classifier constructions cross-linguistically (e.g., Aronoff et al., 2003) and have compared signers with non-signing gesturers (e.g., Schembri et al., 2005), a relatively small number (in addition to those already cited) have examined this morphosyntactic distinction in ASL and other sign languages (Schick, 1987; Kegl, 1990; Janis, 1992; Brentari et al., 2001; Zwitserlood, 2003; and Pfau and Steinbach, 2006).

^7^We use the handshape font (e.g., ) to indicate handshapes. When used for individual handshapes, the image is a picture of a particular hand. When this font is used to represent finger groups, the image stands for a category of handshapes. In these cases, the term “finger group” will precede the image (i.e., “finger group ”), and the image will picture a handshape with extended fingers, without the thumb. For example, the finger group  represents the set of handshapes that includes the range of configurations with the index and middle finger selected: , , , , ,  and .

^8^Handshape complexity can of course be measured along a variety of dimensions, including joint complexity. We focus our attention here on selected finger complexity because the pattern of joint complexity (higher for Handling-HSs than for Object-HSs) does not differ between signers and silent gesturers (Brentari et al., 2012, in preparation), and is therefore not a likely place to observe the development of linguistic structure in emerging languages.

^9^Duality of patterning is not synonymous with phonological organization. Here, the organization of finger group complexity (a type of phonological organization) is isomorphic with the morphological organization, and thus does not show an independent level of organization (i.e., duality of patterning).

^10^This is based on average language-specific, finger group complexity for handshape type; every Object-HS is not more complex than every Handling-HS.

^11^More details about how and to what extent homesigners communicate with the hearing people around them are provided in the descriptions of the child and adult homesigners in this study in the Participants Section.

^12^In two previous studies, hearing gesturers as a group either showed the opposite pattern—higher finger group complexity in Handling handshapes (Brentari et al., 2012)—or very little finger group complexity overall, with no difference between Handling and Object handshapes (Brentari et al., in preparation).

^13^Although unlike Julio, the hearing children have linguistic input and an existing grammatical system, just one that does not use handshape for grammatical functions.

^14^Because we are studying gesture systems in which we cannot assume the existence of a classifier system a priori, henceforth we use the more neutral term “handshape” and refer specifically to Object handshapes (Object-HS) and Handling handshapes (Handling-HS). Likewise, we use the neutral term “event description” for constructions that parallel, in form and function, the classifier predicates of sign languages.

^15^Other studies of the acquisition of classifiers in sign languages include Bernardino, 2006; Tang et al., 2007; however, these studies also do not take up the question of how children begin to use handshapes differently in agentive vs. non-agentive events.

^16^A discussion of the time course of the development of labels is beyond the scope of this paper, but is an active topic of our current work; see Goldin-Meadow et al. (under review) for such a discussion in Nicaraguan adult signers and homesigners.

^17^For two years, there were two young deaf sisters who used Nicaraguan Sign Language at home with their older brother and sister who are Deaf; however, Julio did not attend school regularly during this time.

^18^A subset of these data have been previously published in (Brentari et al., 2012). The analyses reported here replicate and extend those findings by expanding the dataset.

^19^The interlocutor was a native speaker or signer in all but 3 of 14 sessions, and data were collected, according to the parents' preference, at the child's school, in the child's home, or at the regional headquarters of the Deaf Association in Milan (ENS).

^20^Some of the 10 variations of number and orientation described in Figure 4 were represented by multiple trials, thus the overall total number of vignettes was higher than 110 [11 (objects) × 10 (variations)]. For example, variation #5 for the object airplane had two versions of movement without an Agent: one in which the airplane fell off the edge of a table, and one in which the airplane (a wind-up mechanical toy) tumbled over itself. For some objects (e.g., pen), variations #8 and 9 showed the agent picking up the objects (vs. putting them down).

^21^The Agentive events in our stimulus set all involve manipulation of an object by a human agent. Objects can of course be manipulated by other objects, for example, tools. In describing such an event, in which a tool is manipulated by a human agent, it would most likely be represented by signers using a Handling handshape in our classification system.

^22^Julio's brother, who is his primary communication partner using the homesign, was usually in school during our testing sessions with Julio and was therefore unavailable to serve as his interlocutor.

^23^Gestures that were not labels or event descriptions, such as gestures indicating the number of objects, were classified as “Extra Information” and were not coded further.

^24^There are three planes in the signing space: the horizontal plane, the vertical plane, and the mid-sagittal plane (Brentari, 1998).

^25^These criteria for categorizing a particular gesture or sign according to its function as a label or as an event description are reliable in the context of this task, and we make no claim that these criteria would be appropriate to distinguish nouns and verbs across an entire sign language.

^26^For the adult homesign data, interrater reliability for classification of handshapes according to configuration and for classifying handshapes according to type was 90% (Brentari et al., 2012). (Labels were not coded for those analyses).

^27^Three of Julio's original, complete videotaped responses have been annotated and may be viewed online (see Coppola, 2014).

^28^These four objects represent a range of object properties with respect to shape, size and complexity of features, which we expected would affect the handshape configurations that would be used to describe them. For this reason, they were also the objects that were already analyzed for the adult homesigners and thus available for comparison.

^29^Visual inspection of the patterns across sessions did not warrant a statistical analysis of the developmental trajectory: for No-Agent events Julio consistently has no strong preference for handshape type, and for Agent events, he consistently strongly prefers Handling-HSs.

^30^The bar for the child homesigner shows handshapes produced in response to three of the four objects, because he did not produce any Object-HSs for vignettes involving pens.

^31^Julio's inventory of handshapes in the earlier sessions not shown in these charts (7;4, 7;10–8;5, and 9;11) did not differ from those produced by the hearing, silent-gesturing children.

^32^As previously described, we interpret this distribution of finger group complexity as evidence of Julio's developing phonological system. Evidence of phonological development is of course also found in simple lexical items, such as numbers, nouns, and verbs; these uses can and do precede complexity in those same handshapes when they are produced as classifier handshapes (Kantor, 1980). A comparison of the relative timing of these developments in Julio is beyond the scope of this paper; however, we are currently analyzing the handshapes he produces to label objects (comparable to nouns).

^33^The theoretical model for this morphosyntactic contrast is laid out in Benedicto and Brentari (2004). In practice, we have used the performance of native-signing adult signers as criteria for this contrastive use of handshape type in the grammar of established sign languages; while adult signers demonstrate a clear contrast in their use of handshape type, they do not uniformly produce the expected forms 100% of the time (Brentari et al., 2012), underscoring the importance of obtaining behavioral data that converges with theoretical predictions.

^34^Though see Goldin-Meadow et al. (1994) regarding a noun-verb distinction in child homesigners, Goldin-Meadow and Mylander (1990) on gesture order and other patterns in child homesigners from 1;4 to 5;9; Morford (2003) for a longitudinal study of the use of handshape to express motion events in two adolescent homesigners who were recently immersed in ASL. Berk and Lillo-Martin (2012) analyzed the two-word utterances produced over time by two deaf children who began acquiring ASL in an immersion context at the ages of 5;9 and 6;0.

^35^One alternative hypothesis is that the salience of human agents accounts for Julio's use of Handling-HSs. Julio may have inferred that someone put the objects in the frame though the agent was not observed doing so. While we cannot definitively rule this out, neither signers nor hearing individuals gesturing silently in the US and Italy did this; in fact, they used Object-HSs more often for Agentive vignettes than the reverse pattern exhibited by Julio. A second alternative explanation is his young age. Kaplan (1968) found that children asked to show how they would use non-present objects did not robustly use handling gestures until they were 12 years old, suggesting that Julio's early use of Handling-HSs in Agentive contexts does not reflect “default” imitation or an immature pattern.

^36^Though also see anecdotal examples such as the following, from Feldman (1975) (and others discussed by Meier, 1982), in which a child homesigner developed a gesture referring to ice cream that used licking as its iconic base, and continued to use that form even in situations in which the ice cream was in a bowl, and no licking was involved.
