# Supplementary material for: From iconic handshapes to grammatical contrasts: longitudinal evidence from a child homesigner
Source: Front Psychol. 2014 Aug 21;5:830. doi: 10.3389/fpsyg.2014.00830 (PMC4139701; doi:10.3389/fpsyg.2014.00830)

Complete list of stimulus items presented to and responded to by Julio, the child homesigner, at each testing session. Variations 1-5 did not feature an agent (non-agentive): 1-4 were static photographs and variation 5 was a video of the object moving without an agent (typically falling off a table). Variations 6-10 did feature an agent and were short video clips. Items that were represented by multiple variations are labeled a, b, c (e.g., variation 10 for the object cigar was represented by three different videos, labeled 10a, 10b, and 10c). Items that were presented to Julio but did not elicit a usable response are marked with an “x”. Items for which Julio produced a response that could be coded are indicated with a dot (•). Recall that the 7;10 and 8;5 sessions were combined due to the relatively small number of items presented during the 7;10 session.

**Object**	**Variation**	**Session** (age in years; months)					
		**7;4**	**7;10**	**8;5**	**9;11**	**11;4**	**12;8**
book	1	•		x	x	•	•
book	2	x		•		•	•
book	3	x		•		•	•
book	4	x		•		•	•
book	5	x		•	x	•	•
book	6	•		•	•	•	•
book	7	•		•		•	•
book	8	•		•		•	•
book	9	•		•		•	•
book	10	x		•		•	•
cigar	1	x		x	x	•	•
cigar	2	x		x		•	•
cigar	3	x		x		•	•
cigar	4	x		x		•	•
cigar	5	•		•	•	•	•
cigar	6	•		•	x	•	•
cigar	7	•		•		•	•
cigar	8	•		•		•	•
cigar	9	•		x		•	•
cigar	10a	•		•		•	•
cigar	10b	•		•		•	•
cigar	10c	•		•		•	x
coin	1	x	x		•	x	x
coin	2	x	•		•	•	•
coin	3	•	x		•	x	•
coin	4	•	x		•	x	•
coin	5	•	•		•	•	•
coin	6	•	•		•	•	•
coin	7	x	•		x	•	•
coin	8	•	•		x	•	•

Object	Variation	Session (age in years; months)					
		**7;4**	**7;10**	**8;5**	**9;11**	**11;4**	**12;8**
coin	9	x	•		•	•	•
coin	10a	•	•		•	•	•
coin	10b	•	•		•	•	•
lollipop	1	•	x			•	•
lollipop	2	x	x		•	•	•
lollipop	3	x	x		•	•	•
lollipop	4	•	x		•	•	•
lollipop	5	•	•		•	•	•
lollipop	6	•	x		•	•	•
lollipop	7	•	x		•	•	•
lollipop	8	x	•		•	•	•
lollipop	9	x	•		•	•	•
lollipop	10	•	•		•	•	•
marble	1	x		x	x	•	•
marble	2	x		•		•	•
marble	3	•		•		•	•
marble	4	•		•		•	•
marble	5	•		•	x	•	•
marble	6	•		•	•	•	•
marble	7	•		•		•	•
marble	8	•		•		•	•
marble	9	•		•		•	•
marble	10	•		•		•	•
pen	1	x	x	x		•	•
pen	2	•	x	x		•	•
pen	3	•	•	•		•	•
pen	4	•	•	•		•	•
pen	5	•	•	•		•	•
pen	6a	x	•	•		•	•
pen	6b	•	•	•		•	•
pen	7	•	•	•		•	•
pen	8a	•	x	•		•	•
pen	8b	x	x	•		•	•
pen	9	•		•		•	•
pen	10	•	•	•		•	•
plane	1	x			x	x	•
plane	2	•			x	•	•
plane	3	x			•	•	•
plane	4	x			•	•	•

**Object**	**Variation**	**Session** (age in years; months)					
		**7;4**	**7;10**	**8;5**	**9;11**	**11;4**	**12;8**
plane	5a	•			•	•	•
plane	5b	x			•	•	•
plane	6	x			•	•	•
plane	7	•			•	•	•
plane	8	x			•	•	•
plane	9	•			•	•	•
plane	10	•			•	•	•
string	1	x		x	•	•	•
string	2	•		•		•	•
string	3	•		x		•	•
string	4	•		x		•	•
string	5	•		•	•	•	•
string	6	•		•	•	•	•
string	7	•		x		•	•
string	8	x		•		•	•
string	9	x		•		•	•
string	10a	•		•		•	•
string	10b	•		•		•	•
tape	1	•		x	x	•	•
tape	2	•		x		•	•
tape	3	x		•		•	•
tape	4	x		•		•	•
tape	5a	x		•	•	•	
tape	5b	•		x			•
tape	6	•		•	x		•
tape	7	•		•		•	•
tape	8	•		•		•	•
tape	9	•		•		•	•
tape	10	•		•		•	•
tv	1	•		x	x	•	•
tv	2	•		x		•	•
tv	3	x		•		•	•
tv	4	x		•		•	•
tv	5	•		x	•	•	•
tv	6	x		•	•	•	•
tv	7	x		•		•	•
tv	8	•		•		•	•
tv	9	•		•		•	•
tv	10	•		•		•	•

Object	Variation	Session (age in years; months)					
		7;4	7;10	8;5	9;11	11;4	12;8
tweezers	1	•		x		•	•
tweezers	2	•		x		•	•
tweezers	3	•		•		•	•
tweezers	4	x		•		•	•
tweezers	5	•		x	•	•	•
tweezers	6	•		x	x	•	•
tweezers	7	•		x		•	•
tweezers	8	•		•		•	•
tweezers	9	•		•		•	•
tweezers	10	•		•		•	•

Distribution of finger group complexity by presence of an Agent. This chart presents the same data shown in Figure 10 in the main text, separated into vignettes with and without an Agent. "Violations" of the established sign language pattern exhibited the same patterns of finger group complexity as the handshapes that conform to the sign-like pattern. Specifically, in the last two sessions, the Handling-HSs in No-Agent contexts (black bars, top chart) are lower than the corresponding gray bars showing the finger group complexity of the Object-HSs; further, the Object-HSs in Agent contexts (gray bars, bottom chart) are higher than the corresponding black bars showing the finger group complexity of the Handling-HSs. (Finger group complexity was averaged across all 11 object types (e.g., airplane, book, cigar, etc.).)

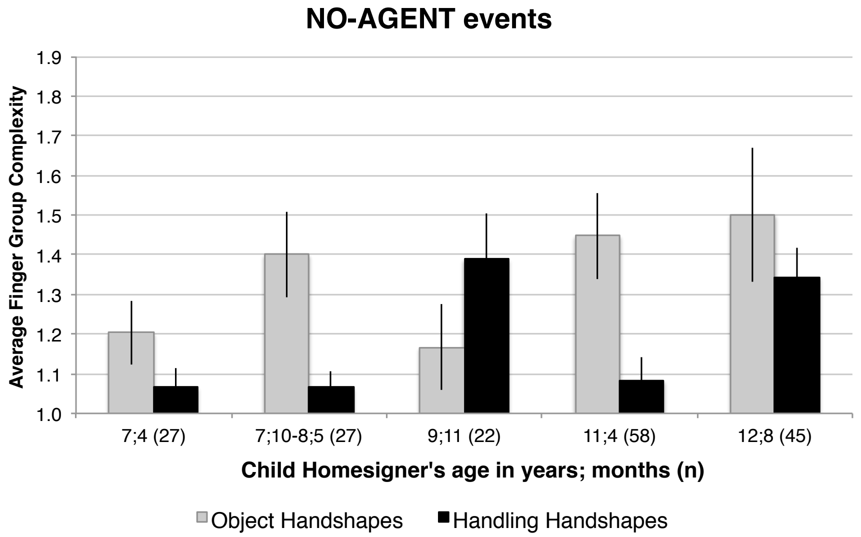

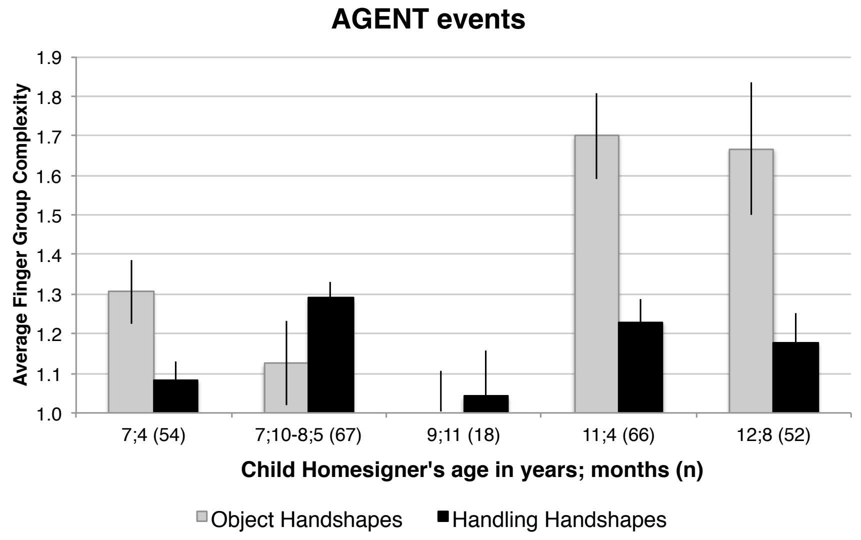

Supplement: Supplementary file 1 [file DataSheet1.PDF]
